# Impacts of the Conductive Networks on Solid‐State Battery Operation

**DOI:** 10.1002/anie.202511534

**Published:** 2025-08-05

**Authors:** Shimao Deng, Yixian Wang, Tianxiao Sun, Wenlong Li, Mingyuan Ge, Jian Wang, Peter Cloetens, Piero Pianetta, David Mitlin, Yijin Liu

**Affiliations:** ^1^ Materials Science and Engineering Program Walker Department of Mechanical Engineering and Texas Materials Institute The University of Texas at Austin Austin TX 78712 USA; ^2^ National Synchrotron Light Source II Brookhaven National Laboratory Upton NY 11973 USA; ^3^ Canadian Light Source Inc. University of Saskatchewan Saskatoon Saskatchewan S7N 2 V3 Canada; ^4^ European Synchrotron Radiation Facility Grenoble 38000 France; ^5^ Stanford Synchrotron Radiation Lightsource SLAC National Accelerator Laboratory Menlo Park CA 94025 USA

**Keywords:** All‐solid‐state batteries, Li^+^/e^−^ channels, Micromorphology, Microscopic electrochemical polarization, Multimodal X‐ray spectro‐microscopy

## Abstract

The micromorphology of composite cathodes is known to play a vital role in determining all‐solid‐state battery (ASSB) performance. However, much of our current understanding is derived from empirical observations, lacking a deeper mechanistic foundation. The “rocking chair” concept of battery chemistry requires maintaining charge neutrality, emphasizing the necessity of examining electrode micromorphology from the perspective of conductive networks. This study systematically investigates the microscopic electrochemical impacts of conductive network micromorphology by varying the Li^+^‐to‐e^−^ channel ratio in cathodes comprising LiNbO_3_‐coated LiNi_0.8_Co_0.1_Mn_0.1_O_2_, Li_6_PS_5_Cl, and carbon fibers. Utilizing multiscale synchrotron‐based spectro‐microscopy, we unravel that unbalanced Li^+^ and e^−^ conducting channels intensify charge polarization within active cathode particles and accelerate their degradation. A further model system with X‐ray nano‐tomography resolved e^−^ and Li^+^ channels indicates that spatially uniform and well‐paired Li^+^ and e^−^ conducting channels are highly desirable as they could promote more uniform lithiation/delithiation, mitigating microscopic electrochemical polarization. Electrode‐scale X‐ray holotomography analysis reveals that the impact of conductive networks is particle‐size‐dependent, with smaller cathode particles being more significantly affected. These findings provide mechanistic insights into the interplay between conductive networks and all‐solid‐state battery operation, laying the groundwork for rational design and optimization of cathode architectures in future solid‐state battery technologies.

## Graphical abstract

The solid‐state composite cathode's hierarchical architecture, characterized by uneven distributions of Li^+^ and e^−^ conductive networks, critically impacts ASSB operation. Here, using model systems with varying Li^+^/e^−^ conductive networks and multimodal spectro‐microscopy, we reveal that unbalanced and unpaired distribution of Li^+^/e^−^ channels across NMC particle surface governs cathode degradation by inducing microscopic electrochemical polarization.

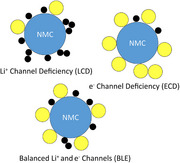

## Introduction

All‐solid‐state batteries (ASSBs) are considered a promising next‐generation energy storage technology due to their potential for high energy density and enhanced safety.^[^ ^1^, ^2^, ^3^, ^4^, ^5^, ^6^ ^]^ One of the crucial elements enabling these advantages is the solid‐state electrolytes (SSEs) that are nonflammable and compatible with Li metal anodes.^[^ ^7^ ^]^ To achieve high performance, ASSBs require SSEs with excellent Li^+^ conductivity, typically exceeding 1 mS cm^−1^ at room temperature.^[^ ^8^, ^9^ ^]^ Through enormous effort, significant progress has been made in developing SSEs with sufficient Li^+^ conductivity, including oxides^[^ ^10^, ^11^, ^12^ ^]^ sulfides,^[^ ^13^, ^14^, ^15^ ^]^ and halides.^[^ ^16^, ^17^, ^18^ ^]^ Beyond efficient Li^+^ transport across the SSE separator, effective Li^+^ and e^−^ transport within the cathode composite is also crucial for ASSB operation.^[^ ^19^, ^20^ ^]^ This necessitates adequate conducting pathways for Li^+^ and e^−^ around the active cathode materials, which heavily depends on the conductive networks within the composite cathode.^[^ ^21^, ^22^, ^23^, ^24^, ^25^ ^]^ Due to the solid nature of SSEs, conductive networks for Li^+^ and e^−^ in cathodes of ASSBs are much more complex and tortuous compared to their counterparts in liquid electrolyte‐based batteries,^[^ ^1^, ^26^, ^27^ ^]^ posing a significant limitation on the battery performance. To design the architecture of conductive networks rationally, a comprehensive understanding of their impacts on ASSB operation is essential.

A typical composite cathode in ASSBs comprises active materials, SSEs, and conductive additives. SSEs and conductive additives form the conductive networks surrounding active materials, enabling Li^+^ and e^−^ transport. Unlike in conventional batteries, where active materials are well integrated with liquid electrolyte and carbon/binder domain (CBD), the solid–solid interfaces in ASSBs restrict interfacial contact, limiting the connectivity between active materials and the conductive networks.^[^ ^28^ ^]^ This results in a highly heterogeneous microstructure with uneven distribution of Li^+^ and e^−^ conducting channels, thereby impeding charge carrier transport and reducing active material utilization.^[^ ^29^, ^30^ ^]^ Furthermore, such heterogenous structure could induce unbalanced Li^+^ and e^−^ transport, which hinders Li^+^ insertion and extraction, accelerates stress accumulation in the active cathode materials, and, ultimately leads to interparticle detachment and intraparticle cracking.^[^ ^31^, ^32^ ^]^ Hence, rational cathode architecture design is critical for high‐performance ASSBs. Given the well‐recognized importance of micromorphology in cathode functionality,^[^ ^33^ ^]^ various strategies have been explored to regulate it, including composition tuning,^[^ ^22^ ^]^ particle size control,^[^ ^34^ ^]^ solvent‐assisted processing,^[^ ^23^ ^]^ interfacial structure tailoring,^[^ ^35^ ^]^ and modulation of component hardness.^[^ ^36^ ^]^ Although these approaches can enhance the performance of ASSBs to some degree, a considerable gap remains relative to liquid electrolyte‐based systems. Bridging this gap requires addressing the fundamental challenge of maintaining sufficient and uniform contact between rigid solid components within the cathode to ensure the efficient Li^+^ and e^−^ transport. A deeper mechanistic understanding of how conductive networks influence such interfacial contact and Li⁺/e^−^ transport in ASSBs can provide insights to better define optimization targets. Previous insights are predominantly derived from macroscopic electrochemical measurements and empirical observations, which capture the average effects but are often insufficient due to the high heterogeneity within the ASSB cathodes. Although some microscopic investigations have emerged, they have primarily focused on Li^+^ conducting network formed by SSEs or the connectivity among cathode particles,^[^ ^37^, ^38^, ^39^, ^40^ ^]^ while the impact of conductive carbon forming an e^−^ conducting network has remained largely unexplored. This oversight limits our understanding of how coupled ionic and electronic transport governs the local electrochemical performance in composite cathodes. Thus, advancing the field calls for deeper investigations incorporating microscopic‐level understanding of both e^−^ and Li^+^ transport mechanisms within cathode composite.

In this work, to investigate the impacts of cathode micromorphology, particularly the configuration of conductive networks, on ASSB operation, we systematically tune the conductive networks by adjusting the Li^+^‐to‐e^−^ channel ratio. Cathodes with Li^+^ channel deficiency (LCD) are nonfunctional, while those with e^−^ channel deficiency (ECD) exhibit more severe polarization and lower utilization compared to the cathodes with balanced Li^+^ and e^−^ (BLE) channels. To further investigate the underlying mechanism, we performed synchrotron‐based scanning transmission X‐ray microscopy (STXM) and transmission X‐ray microscopy (TXM) to analyze charge distribution at both primary and secondary particle scales. Significant intra‐ and inter‐particle charge heterogeneity and structural degradation are observed in cathodes lacking sufficient e^−^ conducting channels, in contrast to BLE counterparts. To further elucidate the impact of conductive networks, a model system using X‐ray‐resolved graphite as the e^−^ conductor was developed to investigate the role of conducting network structures, demonstrating that the heterogeneity and imbalance of Li^+^ and e^−^ channels surrounding cathode active materials are critical determinants of particle‐scale electrochemical polarization. These results highlight the necessity of paired Li^+^ and e^−^ conducting contacts for optimal ASSB operation. We further develop a diffusion distance model to quantify this effect, showing that the impacts of Li^+^ and e^−^ conductive networks on ASSB performance are coupled. Moreover, our study reveals that the size of active cathode particles significantly impacts connectivity between active particles and conducting networks. Although conventional wisdom suggests that small particles in liquid electrolyte‐based batteries are favorable for reaction kinetics due to their shortened Li^+^ diffusion distance,^[^ ^41^, ^42^ ^]^ we observe that small active cathode particles in our ASSB system are not fully electrochemically active. This difference primarily arises from the distinct nature of conductive network formation in ASSB system compared to liquid‐based batteries. In ASSBs, the constraints of solid–solid physical contact prevent small particles with limited surface area from establishing sufficient e^−^ and Li^+^ dual connectivity, particularly when e^−^ channels are randomly and sparsely distributed within cathode of ASSBs. This highlights the necessity of simultaneous and balanced access to both Li^+^ and e^−^ conduction pathways. This work sheds light on the role of conductive networks within cathodes in governing the performance of ASSBs, underscoring the significance of designing composite cathode architecture in the manufacturing of next‐generation ASSBs.

## Results and Discussion

### Solid‐State Cathodes with Varying Li^+^ and e^−^ Conductive Networks

**Figure 1:**
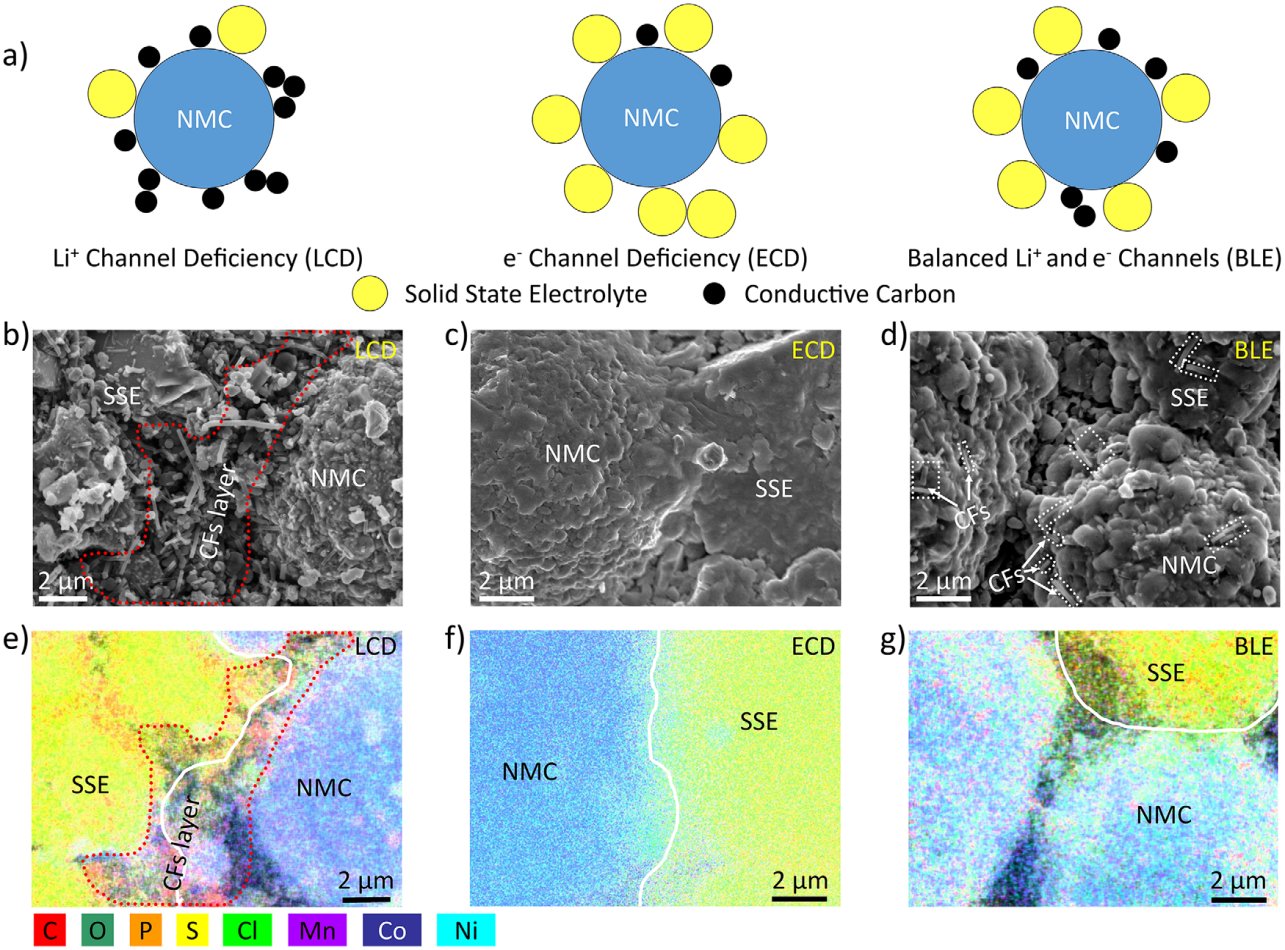
Schematic illustration and micromorphology of heterogeneous cathodes. a) Schematic illustration of cathode particles with varying Li^+^ and e^−^ conducting channels: Li^+^ channel deficiency (LCD), e^−^ channel deficiency (ECD), and balanced Li^+^ and e^−^ (BLE) channels; b)–g) the top‐down SEM images and EDS of surface morphologies of fabricated LCD (b and e), ECD (c and f), and BLE (d and g) electrodes.

The solid‐state composite cathode, consisting of active cathode particles, SSEs, and conductive carbons, exhibits significant heterogeneity. For an individual active cathode particle to effectively participate in the charging–discharging process, it must be simultaneously connected to both Li^+^ and e^−^ conducting channels (Figure S1). However, Li^+^ and e^−^ channels in cathodes of ASSBs can be highly inhomogeneous, placing each individual active cathode particle into a unique microenvironment with varying Li^+^ and e^−^ conductivities, resulting in diverse electrochemical behavior. These microenvironments can be categorized as Li^+^ channel deficient (LCD), e^−^ channel deficient (ECD), or balanced Li^+^ and e^−^ (BLE) channels, as schematically illustrated in Figure 1a. Accordingly, to investigate the microscopic impacts of Li^+^ and e^−^ transport pathways on ASSB operation, three model composite cathodes with controlled micromorphology were fabricated and are referred to as LCD, ECD, and BLE, respectively. These high‐loading cathodes (∼35.4 mg cm^−2^) were prepared using well‐mixed LiNbO_3_‐coated LiNi_0.8_Co_0.1_Mn_0.1_O_2_ (LNO@NMC811, donated NMC), Li_6_PS_5_Cl (LPSCl, the SSE used in this work), and carbon fibers (CFs) in mass ratios of 7:3:1 (LCD), 7:3:0 (ECD), and 7:3:0.3 (BLE), respectively. The BLE formulation, consistent with ratios reported in previous literature,^[^ ^43^, ^44^ ^]^ provides a favorable balance of Li⁺ and e^−^ channels among three model ASSB cathodes. The top‐down scanning electron microscopy (SEM) reveals distinct surface morphologies across three electrodes, demonstrating that NMC particles exhibit varying degrees of contact with surrounding SSEs and CFs. In LCD, CFs are abundant, with some agglomeration at the interface between NMC and SSE, potentially impeding the Li^+^ transport (Figures 1b,e and S2A). In contrast, in ECD, composed solely of NMC and SSE, the only e^−^ conductive channels are through the adjacent NMC particles (Figures 1c,f and S2B). In BLE, CFs are randomly and sparsely distributed, forming more efficient e^−^ channels alongside NMC particles without disrupting Li^+^ conductive channels at NMC‐to‐SSE contacts (Figures 1d,g and S2C). Furthermore, as shown in Figure S3, the ionic and electronic conductivities of three cathode composites are experimentally determined using documented procedures.^[^ ^45^, ^46^ ^]^ The e^−^ conductivity (*σ*_e_) of LCD, ECD, and BLE is 4.99 × 10^−2^, 4.63 × 10^−5^, and 9.56 × 10^−4^ S cm^−1^, respectively. The corresponding Li^+^ conductivity (*σ*_ion_) is 4.86 × 10^−6^, 1.42 × 10^−4^, and 6.21 × 10^−5^ S cm^−1^. LCD exhibits the highest *σ*_e_ but the lowest *σ*_ion_, while ECD demonstrates the highest *σ*_ion_ but the lowest *σ*_e_. BLE achieves a balanced *σ*_ion_ and *σ*_e_, leading to an optimal ASSB performance among the three.

To investigate the impacts of conductive networks on electrochemical performance, both cycling stability and rate capability of the model cathodes were evaluated using polyether ether ketone (PEEK) cells with a two‐electrode configuration. Electrochemical cycling results show that LCD, with a Li^+^ channel deficiency, is nonfunctional (Figure S4A), confirming the necessity of adequate Li^+^ conducting channels for ASSBs operation. In contrast, ECD and BLE exhibit reversible capacities of 191/146 and 209/155 mAh g^−1^ during the initial cycle, corresponding to initial coulombic efficiencies (ICE) of 76% and 74%, respectively (Figure S4B,C). Upon increasing current to 0.33C, BLE maintains an average capacity of 118 mAh g^−1^, outperforming ECD, which delivers only 90 mAh g^−1^ (Figure S5). This improved performance is attributed to the well‐established e^−^ conductive network within BLE, allowing for a more complete electrochemical reaction and electrode utilization. The observed trend is further supported by the rate performance. ECD delivers reversible capacities of 142, 107, 93, and 72 mAh g^−1^ at 0.1, 0.2, 0.3, and 0.5C, respectively. In comparison, BLE achieves 141, 121, 110, and 92 mAh g^−1^ under the corresponding C‐rates. BLE consistently demonstrates a higher capacity, with this advantage becoming more pronounced at higher C‐rates (Figure S6A), and also shows better cycling retention compared to ECD (Figure S6B). To further quantify performance differences, the voltage hysteresis at each current density was calculated based on the voltage difference at 50% state of charge (SOC). The voltage hysteresis values for ECD are 0.12, 0.36, 0.45, and 0.59 V at 0.1, 0.2, 0.3, and 0.5C, consistently higher than those of BLE (0.06, 0.17, 0.24, and 0.36 V at the same current densities, see Figure S7A). The voltage hysteresis gap between ECD and BLE widens with increasing cycling rate (Figure S7B). These results reveal that cell‐level electrochemical polarization is more significant in ECD, suggesting that insufficient e^−^ conducting channels have led to sluggish reaction kinetics in ECD.

### Charge Distribution Within Primary and Secondary Cathode Particles

**Figure 2:**
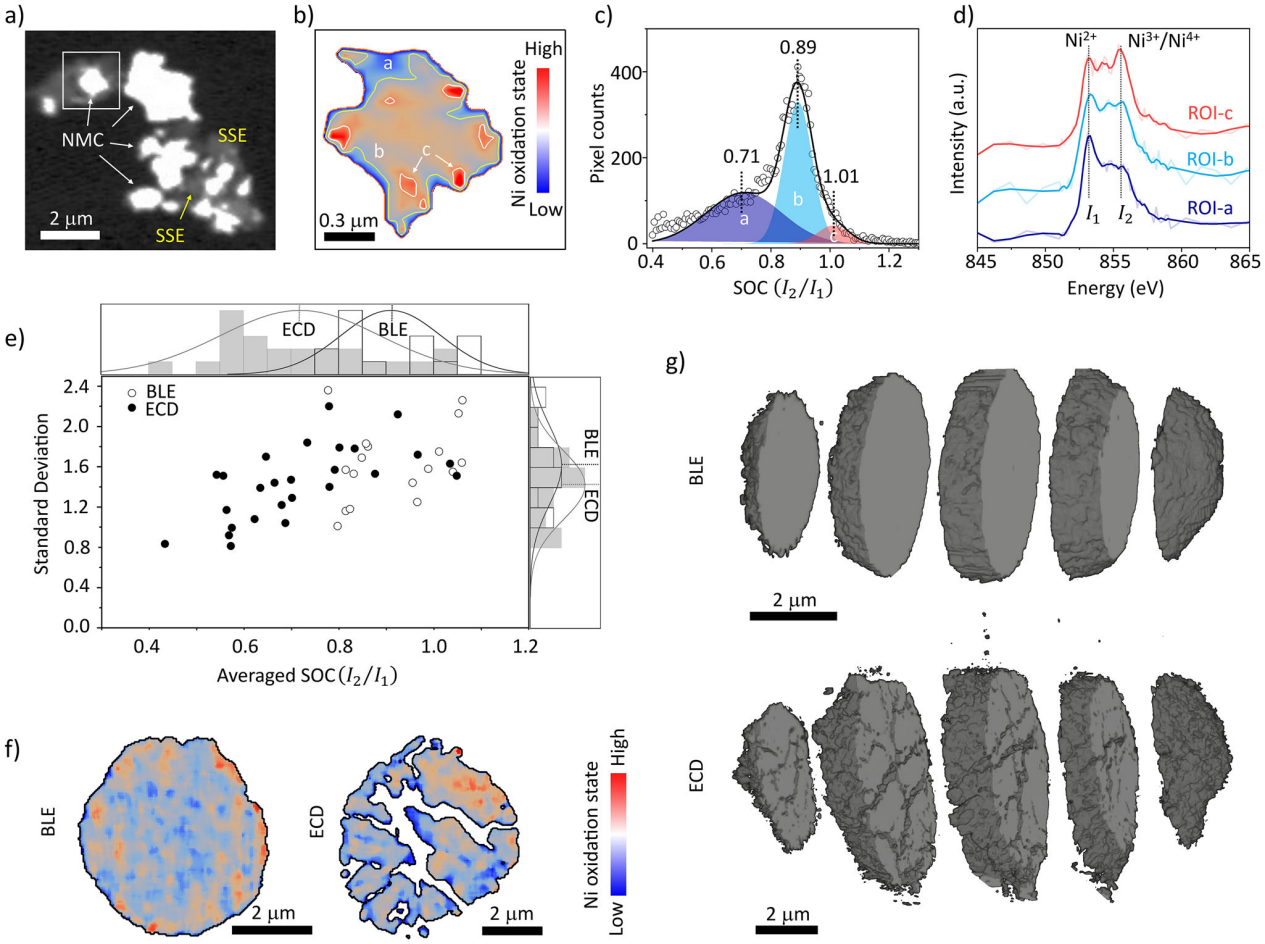
Charge distribution within primary and secondary NMC particles harvested from cycled cathode at discharged state. a) STXM image of cycled BLE primary particles; b) Ni oxidation state mapping within a primary particle selected from (a); c) Probability distribution of Ni oxidation state within the selected primary particle; d) Ni L_3_‐edge XAS spectrum extracted from the regions of interest: a–c in (b); e) Statistical analysis of Ni oxidation state and heterogeneity for over 20 primary particles of BLE and ECD, respectively; f) Charge distribution within secondary particles collected from cycled BLE and ECD. The boundaries of particles are outlined with black contours; g) 3D structure of randomly selected secondary particles of BLE and ECD.

To gain further insights into the underlying mechanisms for the observed cell‐level polarization, chemically sensitive X‐ray microscopy was conducted to probe the charge distribution within active cathode particles harvested from cycled ASSBs at the discharged state. Owing to its high spatial resolution, STXM was employed to investigate the primary particles,^[^ ^47^ ^]^ the smallest crystalline units typically in the nanometer or micrometer range that make up larger aggregates. As shown in Figure 2a, primary particles from the BLE sample, ranging from a few hundred nanometers to around 2 µm in size, are dispersed within the conductive networks. Notably, CFs with a diameter of approximately 100 nm are beyond the resolution limit of STXM due to the low X‐ray absorption contrast and are therefore not visualized here. In NMC‐based cathode materials, Ni serves as the primary redox‐active element, and its oxidation state is closely tied to the (de)intercalation of Li⁺, making it a reliable indicator of the local state of charge (SOC).^[^ ^48^ ^]^ In the STXM experiment, we focus on the Ni L_3_‐edge, which exhibits two distinct peaks at ∼853 and ∼855.5 eV, respectively. Peak 1 (∼853 eV) is attributed primarily to Ni^2+^, while peak 2 at ∼855.5 eV corresponds to contributions from Ni^3+^ and Ni^4+^. Upon charging and Ni oxidation, the relative ratio between *I*_2_ and *I*_1_ increased.^[^ ^49^, ^50^, ^51^ ^]^ Therefore, the peak intensity ratio (*I*_2_/*I*_1_) is used here to color‐code the Ni valence map across the scanned areas and to qualitatively reflect the state of charge of primary particles. A higher *I*_2_/*I*_1_ratio indicates a higher average Ni valence and a more charged state. Through this approach, significant inhomogeneity in Ni oxidation state is observed among different primary particles (Figure S8), as well as within individual primary particles. A closer inspection of a randomly selected primary particle from the BLE sample shows clear spatial segmentation into regions with distinct SOCs (Figure 2b). The histogram of the *I*_2_/*I*_1_ map, i.e., the peak intensity ratio map, over this particle is shown in Figure 2c, clearly demonstrating three distinct components with different SOCs. To validate this observation, we extract the Ni L_3_‐edge signature from these three regions of interests (ROIs) (Figure 2d). The clear suppression of *I*_2_ from ROIs C to B to A, nicely corroborates the SOC variations observed in Figure 2b.

For statistical significance, we analyzed the STXM images of more than 20 primary particles from both ECD and BLE samples (Figures S8 and S9). For each particle, we calculate the averaged SOC and its standard deviation to quantify the corresponding inter‐/intraparticle heterogeneity. As shown in Figure 2e, ECD exhibits a broader SOC distribution, suggesting that e^−^ channel deficiency aggravates the interparticle charge heterogeneity at primary particle level. This trend aligns with the more severe median voltage hysteresis observed in ECD at the cell level. The standard deviation, reflecting intra‐primary‐particle heterogeneity, shows a similar distribution between BLE and ECD, though the value for BLE is slightly higher, indicating no significant difference in intra‐primary‐particle heterogeneity between the two.

To go beyond the primary particles and further investigate the charge distribution at the secondary particles, which are larger spherical agglomerates of primary particles and are commonly used as building blocks in batteries, TXM was employed.^[^ ^52^, ^53^, ^54^ ^]^ The X‐ray energy scan in these experiments was focused on the Ni K‐edge, which also offers sensitivity to the oxidation states of Ni. While the secondary‐particle‐level SOC heterogeneity prevails in both ECD and BLE samples (Figure 2f), a pronounced charge polarization is observed in the ECD particle, with the upper‐right portion of the particle being more oxidized (red). This secondary‐particle‐level charge polarization is quantified by the offset of the SOC centroid with respect to the particle geometrical centroid. This polarization can be affected by several factors, particularly the imbalance in local conductive networks for Li^+^ and e^−^, which are governed not only by the initial micromorphology of the as‐fabricated electrodes but also by structural evolution during cycling, such as multiscale cracking. The comparison between ECD and BLE clearly indicates that e^−^ channel deficiency plays a crucial role in the microscopic charge polarization effect. The 3D reconstructions of these two particles further reveal a higher degree of structural disintegration in the ECD sample (Figure 2g). Charge polarization and structural disintegration can amplify each other, creating a self‐reinforcing cycle. We acknowledge that the particle‐to‐particle variation can be significant. However, the consistency between this particle‐level observation and the cell‐level electrochemical data nicely supports our narrative.

### Conductive Networks’ Impact on Microscopic Charge Polarization

**Figure 3:**
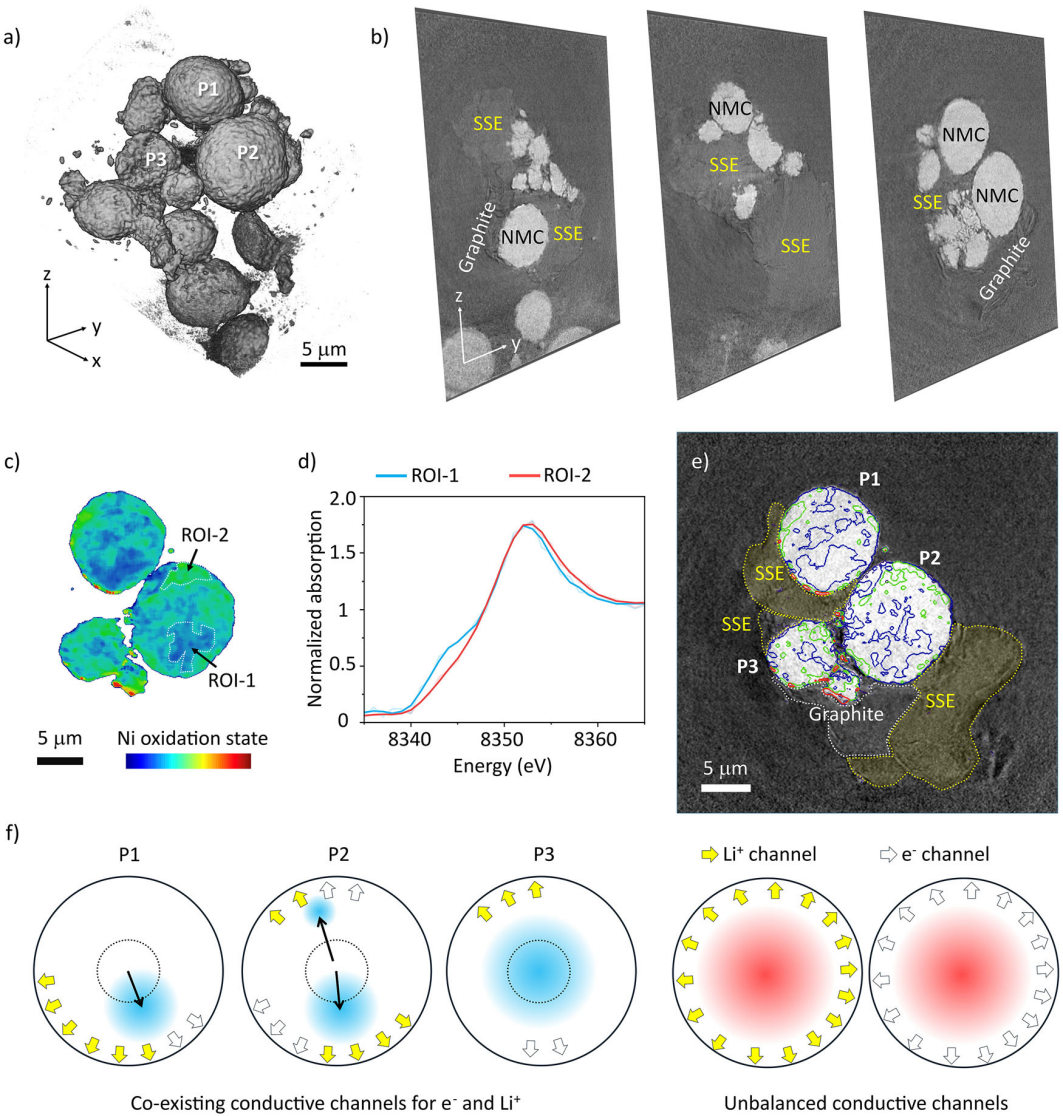
Conductive networks’ impact on the charge polarization at the particle level. a) 3D rendering of the composite cathode consisting of NMC, SSEs, and graphite particles; b) Selected 2D virtual slices through the reconstructed volume; c) Ni oxidation state heterogeneity over a selected 2D virtual slice; d) Ni K‐edge XANES spectra extracted from ROI‐1 and ROI‐2 annotated in (c); e) The contour view of the Ni oxidation state map in (c) and its correlation with the spatial distributions of SSE and graphite; f) Schematic diagrams illustrating the charge polarization affected by the conductive networks’ configuration.

The results presented above suggest that, on the one hand, the microscopic charge heterogeneity is ubiquitous, on the other hand, the main difference between ECD and BLE could be the polarization effect, which is determined by the spatial distributions of Li^+^ and e^−^ conducting channels. Unfortunately, due to the nanoscale dimensions and low X‐ray absorption contrast of CFs, the e^−^ conductive network cannot be directly visualized in the X‐ray imaging results discussed above, hindering an in‐depth mechanistic understanding. To overcome this limitation, we designed a model system with graphite serving as the e^−^ conducting agent due to its larger particle size and enhanced X‐ray absorption contrast, while Li_6_PS_5_Cl remained as solid‐state electrolyte (Figures S10 and S11). Although this model system does not directly correspond to any of the three architectures (BLE, LCD, or ECD), it enables us to directly visualize and distinguish the e^−^ and Li^+^ conductive channels surrounding individual NMC particles. Figure 3a displays the 3D reconstruction of a solid‐state composite cathode composed of NMC, SSEs, and graphite particles, harvested at the fully discharged state. A closer look at the 2D virtual slices in Figure 3b suggests that nano‐tomography offers sufficient resolution and contrast to distinguish structural components based on their morphology and absorption contrast. NMC particles, SSEs, and graphite are clearly differentiated, with graphite exhibiting a characteristic wrinkled structure, while SSE particles appear relatively smooth. Their distinct X‐ray absorption properties further enhance segmentation accuracy (Figure S12).

One virtual 2D slice of model system was selected to further study the correlation between microscopic charge polarization and the local arrangement of the conductive networks (Figure S13). At the fully discharged state, all NMC particles are expected to reach a low state of charge (SOC), corresponding to a low Ni valence. However, as depicted in Figure 3c,d, significant charge polarization persists in these NMC particles, and it is strongly influenced by the spatial configuration of NMC‐to‐SSE and NMC‐to‐graphite contacts (Figure 3e,f). In particle 1 (P1), the lower left corner is in good contact with SSE and the lower right of P1 interfaces with an adjacent NMC particle (P2), which can also serve as the e^−^ conducting channel.^[^ ^55^ ^]^ Consequently, the lower portion of P1 exhibits a lower SOC, indicating this region is more fully lithiated upon cell discharge compared to other regions. Similarly, the lower part of P2, which is in contact with both SSE and graphite, also demonstrates a lower SOC, as does the small region at P2's upper left corner, where it interfaces with SSE and P1. In contrast, particle 3 (P3), which has SSE located at its upper end and graphite at its lower end, respectively, shows minimal polarization. However, its average SOC of P3 is slightly higher, meaning incomplete lithiation upon cell discharge. Additionally, schematic illustrations of extreme scenarios are also provided in Figure 3f. For particles entirely embedded in either SSE or graphite, they will be electrochemically isolated and contribute negligibly to the overall capacity. Notably, the regions with paired Li^+^ and e^−^ channels contact points appear to be kinetically favored, whereas regions in contact with only one type of channel exhibit slower reaction kinetics. This spatial imbalance in conductive network distribution leads to interparticle charge polarization, accelerating cathode degradation. The unbalanced and unpaired distribution of Li^+^/e^−^ channels surrounding NMC particles is a prominent feature in triple‐phase component electrodes in ASSBs. This structural asymmetry induces microscopic charge polarization, contributing to the limited lifespan of ASSBs. This underscores that the objective of structural optimization for ASSBs cathode should be to establish well‐integrated conductive networks with uniformly distributed and paired Li^+^ and e^−^ channel contact points.

**Figure 4:**
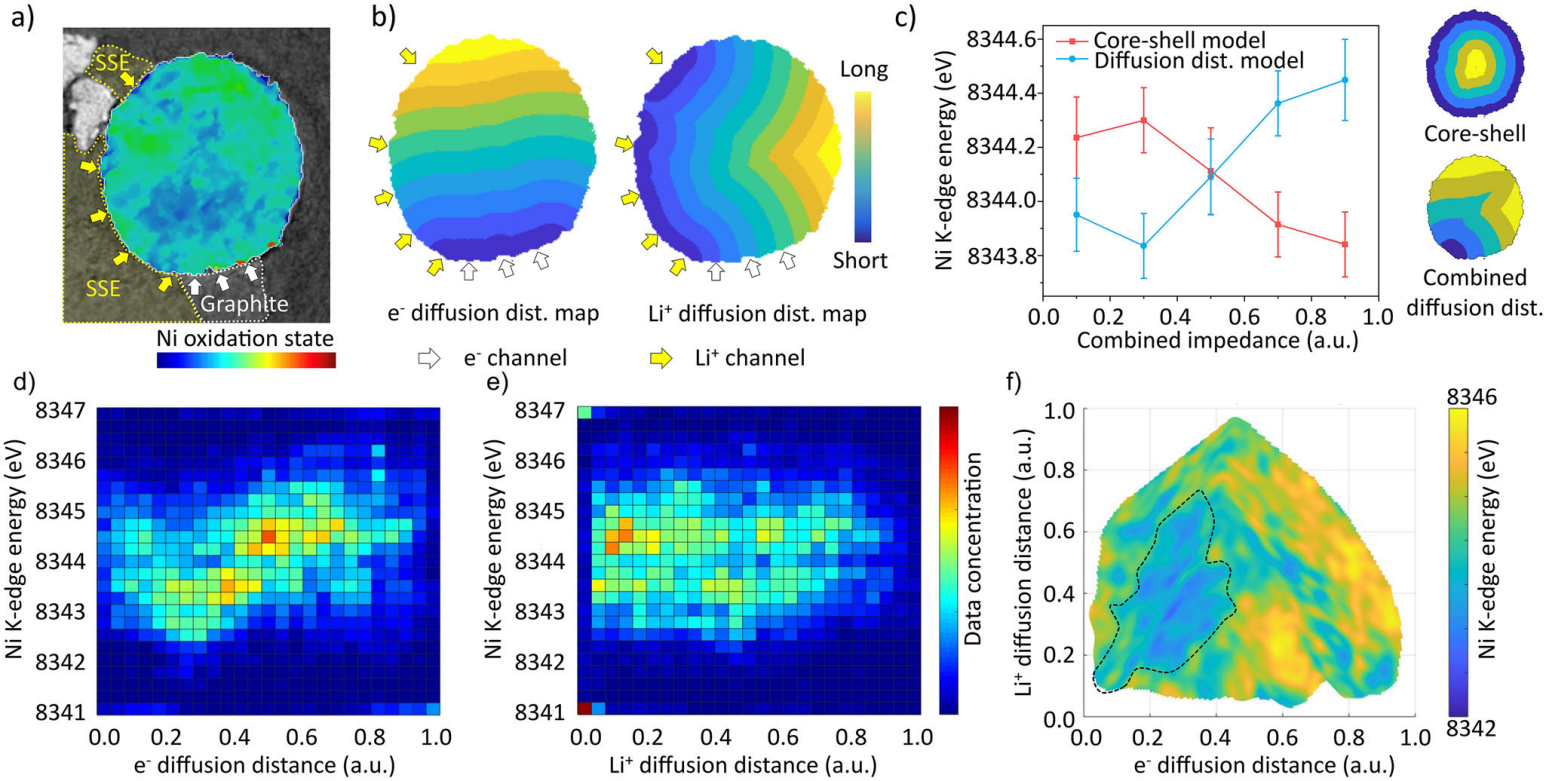
Analysis of conductive networks’ impact on the charge polarization using a combined diffusion distance model. a) Charge mapping and identified conductive network of the selected NMC particle for in‐depth analysis; b) Normalized e^−^ diffusion distance map and Li^+^ diffusion distance map of the selected NMC particle; c) Ni K‐edge energy plotted against combined impedance ([0–0.2], [0.2–0.4], [0.4–0.6], [0.6–0.8], and [0.8–1.0]) from the combined diffusion distance model and core‐shell model, the error bar is defined as one‐tenth of the full width at half maximum (FWHM) of the K‐edge energy distribution for corresponding region; d) Ni K‐edge energy plotted against e^−^ diffusion distance; e) Ni K‐edge energy plotted against Li^+^ diffusion distance; f) 2D map illustrating the relationship between Ni oxidation state and e^−^/Li^+^ diffusion distances. The analyses shown in (d–f) are based on the same NMC particle presented in (a).

As we emphasize the importance of balanced conductive networks, it is critical to point out that the combined e^−^ and Li^+^ diffusion ultimately dictates the functionality of ASSBs. To better understand how e^−^ and Li^+^ conducting channels contribute to the charge polarization, here we developed a concept that evaluates the local reaction impedance within NMC particles based on a combined diffusion distance model. In this model, the Li^+^ diffusion distance is defined as the distance from a certain pixel within the NMC particle to its nearest NMC‐to‐SSE contact point. Similarly, the e^−^ diffusion length can also be determined by searching for the nearest NMC‐to‐graphite contact point. They are used as proxies for the relative transport impedance of the respective charge carriers. Since the electronic conductivity of NMC is significantly lower than that of graphite, electron conduction through NMC‐to‐NMC contacts is not considered in this model. Figure 4a shows an NMC particle in contact with both SSE and graphite at its lower left corner. This particle exhibits significant charge polarization and is selected for further analysis. Using the diffusion distance model, Li⁺ and e^−^ diffusion distance maps were generated (Figure 4b). As previously discussed, a combined and balanced diffusion of Li^+^ and e^−^ would be desirable. To elucidate the inherent correlation, we define the combined transport impedance by integrating Li^+^ and e^−^ diffusion distance of each point within the NMC particle, as derived from the corresponding diffusion distance maps, to estimate local transport resistance, and analyze its correlation with the local SOC. For analysis, the combined impedance of NMC was divided into five regions: [0–0.2], [0.2–0.4], [0.4–0.6], [0.6–0.8], and [0.8–1.0] (Figure S14). As anticipated, Ni oxidation state exhibited an upward trend as the combined impedance increased (Figures 4c and S15). This correlation indicates that the regions with a lower combined impedance can react to a fuller extent during cell operation. For comparison, a core‐shell model commonly employed in liquid‐electrolyte batteries was also applied here (Figures 4c and S16). In this core‐shell model, Ni oxidation state shows a downward trend as the impedance increases, which contradicts the experimental trend. This comparison highlights that conductive network distribution critically governs lithiation/delithiation pathways in solid‐state systems. Unbalanced spatial distributions of Li^+^ and e^−^ channels are likely responsible for the electrochemical polarization across particle scale to cell‐level observed in ASSBs. Furthermore, we attempted to further decouple the roles of e^−^ and Li^+^ impedance by plotting the Ni K‐edge energy against e^−^ diffusion distance and Li^+^ diffusion distance, respectively (Figure 4d,e). As shown in Figure 4d, Ni K‐edge energy and e^−^ diffusion distance demonstrate a rather weak correlation. This correlation is further diminished in Figure 4e. Overall, no clear or consistent trend was observed when either parameter was considered individually. However, when both diffusion distances were considered together, it became evident that regions with lower and balanced combined diffusion impedance exhibit lower local SOC when the cell is fully discharged (Figure 4f). This finding further confirms that Li^+^ and e^−^ channels jointly determine battery performance, both being indispensable, and highlights the significance of their quantitative and spatial balance.

### The Electrochemical Heterogeneity at the Electrode Scale

**Figure 5:**
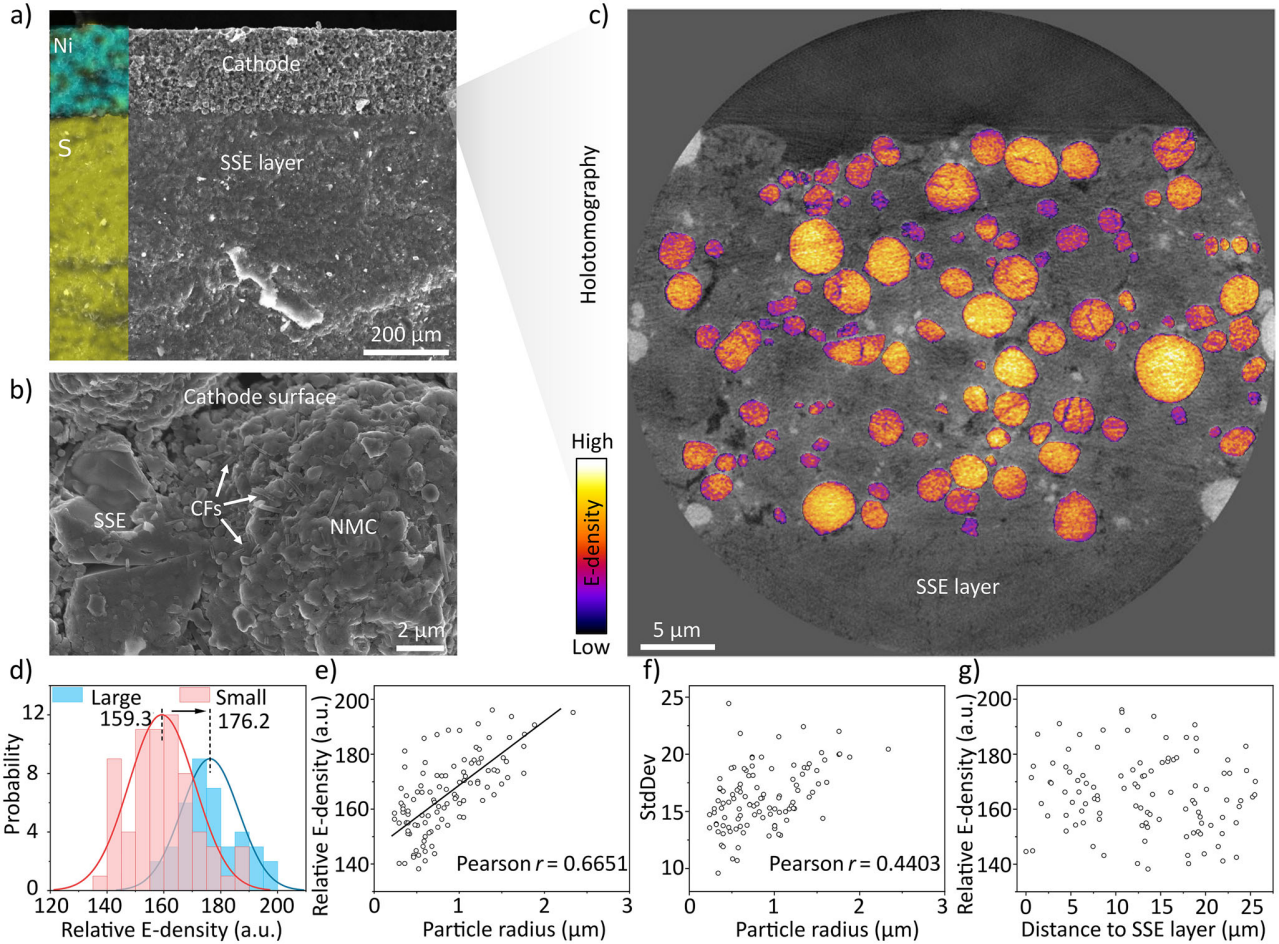
Heterogeneity at the electrode scale of a cathode with balanced Li^+^ and e^−^ channels. a) SEM image of battery cross‐section; b) SEM image zooming in on an individual NMC particle surrounded by SSE and CFs; c) A lateral virtual slice of cathode with the electron density distribution (E‐density) of NMC particles; d) Relative E‐density (reflected by phase‐contrast brightness) distribution in small particles (radius < 1 µm) and large particles (radius > 1 µm); e)–g) Correlation analysis between relative E‐density and particle sizes (e), standard deviation and particle sizes (f), and relative E‐density and depth (g).

At the electrode scale, NMC particles are embedded within SSEs matrix, with CFs sporadically distributed across the surfaces of both NMC and SSE particles, leading to particle‐to‐particle variations in local Li^+^ and e^−^ conductivity (Figure 5a,b). As Li^+^ and e^−^ conducting networks jointly determine the battery performance, such conductive heterogeneity may induce asynchronous and uneven charge–discharge responses among individual particles. To investigate the NMC particles’ behavior at the electrode scale, we employed nano‐resolution hard X‐ray phase‐contrast holotomography, which has been shown to have the capability of quantitatively retrieving the electron density (E‐density),^[^ ^56^ ^]^ a physical property that correlates with SOC of the active cathode materials. In our prior work, we established that the E‐density of NMC increases upon discharging at low SOC,^[^ ^57^ ^]^ which provides a nondestructive and spatially resolved means of probing electrochemical behaviors of a large number of NMC particles within electrode.

As shown in Figure 5c, significant E‐density heterogeneity is observed across the electrode, indicating that NMC particles retain varying SOC after discharge. Notably, E‐density exhibits a size‐dependent trend. To quantify this, NMC particles were segmented (Figure S17) and classified into two groups, the large and small particles, based on their sizes. Statistical analysis reveals that large particles demonstrate higher E‐density distribution than their smaller counterparts (Figure 5d). Further analysis of over 100 NMC particles shows a clear positive correlation between E‐density and particle size, with a Pearson correlation coefficient of 0.6651 (Figure 5e). This suggests that larger particles undergo more complete discharge. As shown in Figure 5c, most NMC particles are well‐embedded in conductive SSE networks with abundant Li^+^ conducting channels. However, CFs are sporadically dispersed (Figure 5b), posing a risk of insufficient contact with small NMC particles due to their limited surface area. Consequently, small particles exhibit relatively more inferior reaction kinetics and incomplete discharge. Nevertheless, some small particles still show high E‐density distribution, likely due to fortuitous contact with both Li⁺ and e^−^ conducting channels. As a result, small particles exhibit a significant interparticle heterogeneity. In contrast, larger particles are more likely to maintain continuous contact with both CFs and SSEs, thus achieving more consistent and complete lithiation when the cell is discharged. Furthermore, the standard deviation of relative E‐density within individual particles is quantified, showing a moderate positive correlation with the particle size (Figure 5f), which indicates larger particles tend to exhibit greater intraparticle heterogeneity, possibly due to internal Li^+^ diffusion limitations. Additionally, to examine depth‐dependent effects, the relative E‐density of each particle was plotted against its distance from the SSE separator (Figure 5g). No clear trend was observed, indicating the absence of significant depth‐dependent SOC heterogeneity in this sample, a common issue in thick electrodes due to limited Li^+^ diffusion.^[^ ^29^ ^]^ Collectively, these results emphasize that, while an optimized macroscopic Li⁺/e^−^ channel ratio enhances overall electrochemical performance, microscopic heterogeneity in conductive network structure and cathode material electrochemistry persists. Across the composite solid‐state cathode, the impact of microscopic morphological heterogeneity is size‐dependent, with smaller active particles being more strongly affected and exhibiting greater electrochemical variability. This finding is different from the traditional cognition of liquid‐electrolyte‐based batteries, where smaller particles exhibit better reaction kinetics.^[^ ^41^, ^42^ ^]^ This discrepancy arises from their fundamental differences in conductive network architecture. In liquid‐electrolyte‐based batteries, active particles are well‐embedded in carbon/binder domain and the porous electrode is thoroughly soaked, so the e^−^ and Li^+^ conductive networks are often not the limiting factors. In contrast, ASSB cathodes depend on the discrete and spatially constrained arrangement of NMC, SSE, and conductive additives, rendering micromorphology a significant factor that governs electrochemical heterogeneity and polarization.

## Conclusion

The conductive networks that govern the micromorphology of composite cathodes play a crucial role in determining ASSB performance. To elucidate the underlying mechanisms at the microscopic level, we designed composite cathodes with systematically varied conductive network structures by adjusting the Li^+^‐to‐e^−^ channel ratio. STXM and TXM analyses revealed that insufficient e^−^ conducting channels lead to significant inter‐ and intra‐particle heterogeneity in primary particles. This further results in pronounced polarization and chemomechanical degradation in secondary particles, aligning well with macroscopic electrochemical polarization observed at cell level. A model system with X‐ray‐resolved Li^+^ and e^−^ channels for investigating impacts of local Li^+^ and e^−^ channels distribution on individual particles unraveled that regions contacted with both Li^+^ and e^−^ conducting channels support efficient reaction kinetics, while other unbalanced regions exhibit sluggish responses, leading to microscopic electrochemical polarization within secondary particles and accelerated structural disintegration. At the electrode scale, the impacts of conductive networks are particle‐size dependent. Smaller particles, with limited surface area, are more likely to suffer from insufficient e^−^ contact and reduced reaction kinetics. These findings highlight the necessity of optimizing cathode micromorphology by controlling active particle size and establishing balanced conductive networks with spatially uniform, paired Li⁺ and e^−^ conducting channels. Strategies such as premixing solid‐state electrolytes with conductive additives, field‐guided assembly, and 3D printing offer promising pathways toward achieving such structural uniformity, ultimately enhancing ASSB capacity and cycling stability and supporting their future commercialization.

## Supporting Information

The authors have cited additional references within the Supporting Information.

## Conflict of Interests

The authors declare no conflict of interest.

## Supporting information



Supporting Information

## Data Availability

The data that support the findings of this study are available from the corresponding author upon reasonable request.
