# Supplementary material for: Impacts of the Conductive Networks on Solid‐State Battery Operation
Source: Angew Chem Int Ed Engl. 2025 Aug 5;64(39):e202511534. doi: 10.1002/anie.202511534 (PMC12455428; doi:10.1002/anie.202511534)
Supplement: Supplementary file 1 — Supporting Information [file ANIE-64-e202511534-s001.docx]

**Supporting Information**

**Impacts of the Conductive Networks on Solid-State Battery Operation**

Shimao Deng^1, #^, Yixian Wang^1, #^, Tianxiao Sun^1^, Wenlong Li^1^, Mingyuan Ge^2^, Jian Wang^3^, Peter Cloetens^4^, Piero Pianetta^5^, [David Mitlin](https://onlinelibrary.wiley.com/authored-by/Mitlin/David)^1, *^, Yijin Liu^1, *^

^1^Materials Science and Engineering Program, Walker Department of Mechanical Engineering and Texas Materials Institute, The University of Texas at Austin, Austin, TX 78712, USA

^2^National Synchrotron Light Source II, Brookhaven National Laboratory, Upton, NY 11973, USA

^3^Canadian Light Source Inc., University of Saskatchewan, Saskatoon, Saskatchewan S7N 2 V3, Canada

^4^European Synchrotron Radiation Facility, Grenoble 38000, France

^5^Stanford Synchrotron Radiation Lightsource, SLAC National Accelerator Laboratory, Menlo Park, CA 94025, USA.

^#^These authors contributed equally

^*^Email: david.mitlin2@utexas.edu (D. M.) & liuyijin@utexas.edu (Y. L.)

**Materials and Methods**

**Electrode Preparation and Cell Assembly**

LiNbO_3_-coated LiNi_0.8_Co_0.1_Mn_0.1_O_2_ (LNO@NMC811, NMC) powder (NEI Corporation), Li_6_PS_5_Cl (LPSCl) (NEI Corporation), and Li metal were used as the cathode materials, solid-state electrolytes (SSEs), and anode, respectively. Cathodes for LCD, ECD, and BLE were prepared by mixing NMC, SSEs, and carbon fibers (CFs, Sigma Aldrich) in the following weight ratios: 7:3:1 for LCD, 7:3 for ECD, and 7:3:0.3 for BLE. The materials were thoroughly ground in a mortar for 30 minutes. For the model system, the cathode was prepared under the same procedure, with a weight ratio of 6:4:2 for NMC811, Li_6_PS_5_Cl, and graphite.

For cell assembly, 150 mg of wet ball-milled LPSCl was first pressed into a pellet using a 12 mm polyether ether ketone PEEK mold at 530 MPa for 1 minute. The milling procedure followed the method described in previous work^[1]^. Next, 40 mg of the prepared cathode material was placed on top of the pellet and pressed at 867 MPa for 2 minutes. A ~100 μm-thick Li metal plate was attached to the other side of the pellet without compressing. Afterwards, the assembled cell was placed into a cell case, tightened using a torque of 20 lb-in (corresponding to 9 MPa pressure) to ensure good contact for electrochemical testing.

Galvanostatic cycling was performed using a Land CT2001A battery tester. LCD, ECD, and BLE were cycled at 0.1 C for the first 2 cycles, followed by 0.33 C for the remaining cycles. The electrochemical measurements were carried out within the voltage window of 2.8 – 4.3 V. The model system using graphite conducting agent was cycled for 14 cycles under the same procedure. All electrochemical measurements were conducted under room temperature (~23 ^o^C).

Ionic and electronic conductivities were measured by DC polarization techniques. The electron-blocking symmetric cell composed of five-layer pellet (Li | SSE | Cathode | SSE | Li) was employed to acquire ionic conductivity. The cathode composites were initially pressed at 530 MPa, followed by pressing the SSE onto both sides at the same pressure. The assembled symmetric cell was then operated under an applied pressure of approximately 10 MPa. Two DC voltages, 0.05 and 0.1 V were used, and corresponding current response was recorded. The three-layer (Stainless | Cathode | Stainless) ion-blocking cell was chosen to measure electronic conductivity. Cathode composites were pressed under 530 MPa and the cell was run under around 20 MPa. The current response was recorded under 0.25 and 0.5 V. The ionic and electronic conductivities were calculated according to the following equation:

$$\text{σ=} \frac{\text{I}}{\text{V}}\text{∙}\frac{\text{L}}{\text{A}}$$

Where I, V, L, and A represent current, voltage, cathode thickness, and cathode area, respectively.

**SEM characterizations**

Scanning electron microscopy (SEM) and energy dispersive spectroscopy (EDS) analyses were conducted using a field emission SEM (Apreo 2C, ThermoFisher Scientific). To prevent air exposure, samples were mounted on an air-sensitive transfer holder within the argon-filled glovebox prior to SEM characterization. The model system and cross-section SEM images were acquired at an accelerating voltage of 30 kV, while other SEM images were collected at 5 kV. The beam current was 0.4 nA, and a secondary electron detector was used. EDS analyses were performed at an accelerating voltage of 20 kV.

**STXM stacking**

The STXM experiments were conducted at the 10ID-1 SM beamline at the Canadian Light Source (CLS). The cathode particles were prepared by scraping them off the electrode surface, dispersing them in xylene, and then drop-casting the suspension onto a SiN window. To prevent air exposure, another SiN window was placed on top, forming a sandwich structure. For STXM stacking, incident X-ray energies were tuned across the Ni *L*_3_-edge, covering the energy range from 845 to 885 eV. Data analysis was performed using the aXis2000 software^[2]^.

**Full-field TXM nano-tomography**

TXM imaging of the cathode materials was conducted at the full-field X-ray imaging (FXI) beamline (18-ID) at NSLS-II, Brookhaven National Laboratory, using a pixel size of 20 nm. The cathode used for TXM is scraped from discharged electrode using a blade inside a glove box, yielding clusters of particles. The blade-scraped clusters retain a relatively intact microenvironment, particularly for particles embedded within the cluster interior. These scraped clusters were then loaded into capillary tubes and sealed with epoxy within the glove box to maintain an inert environment before being transferred to the beamline. In TXM experiments, clusters with well-preserved morphology were selected. Projection images were taken across a 0–180° rotation under fly scan mode. For 3D XANES, incident X-ray energies were tuned across the Ni *K*-edge, covering the energy range from 8210 to 8700 eV with 63 energy points. The TXM data were processed by an in-house developed software package known as TXM-Wizard^[3]^.

**Diffusion distance model**

A Python-based computational model is developed to calculate the diffusion distances of Li⁺ and e⁻ within the NMC particles. The diffusion distance is calculated by determining the geometric distance of each pixel to either the NMC-to-SSE interface or the NMC-to-graphite interface, based on their geometric coordinate. Specifically, the process begins with an image segmentation operation to label the regions of interest. Following that, all pixels at the NMC-to-SSE or NMC-to-graphite boundaries are identified as contact points. The Euclidean distance transform is then applied to compute the shortest distance from each pixel within the NMC particle to the nearest contact points, generating a distance map. Prior to importing the images into the model, manual preprocessing is conducted to validate the segmentation of NMC-to-SSE and NMC-to-graphite interfaces, as well as the boundary of NMC particles. The background pixel intensity was set to 0. The calculated impendence distribution within the NMC particle is normalized to a range of 0-1. Since the ionic/electronic conductivity of different regions within the same NMC is consistent, the relative values of ion/electron impedance within the spatial distribution are not affected by these conductivities. Therefore, this model system disregards the conductivities of Li⁺ and e^-^ in NMC, focusing exclusively on the geometric diffusion distances. For future work, the influence of grain boundaries within the NMC particles should be considered. Automatic high throughput segmentation and intraparticle microstructure awareness could improve the effectiveness of our method.

**Hard X-ray phase contrast holotomography**

Hard X-ray phase contrast holotomography measurements were conducted at the ID16A-NI nano-imaging beamline at the European Synchrotron Radiation Facility (ESRF) in Grenoble, France. This beamline is equipped with a high-energy hard X-ray nano-probe, capable of focusing down to approximately 20 nm with a photon flux of up to 10^12^ photons per second at ΔE/E ~ 1%. The nano-focusing is achieved through two pairs of multilayer-coated Kirkpatrick–Baez (KB) optics, operating at energies of 17 and 33.6 keV, respectively. For our measurements at 17 keV, the sample was positioned downstream from the KB focus, and magnified radiographs were captured using an X-ray detector with a FReLoN CCD camera, with a 2048 × 2048 pixel array. After magnification, the pixel size effectively corresponded to 70 nm. The exposure time per radiograph was set to 0.2 seconds. Each full phase contrast nano-tomography scan involved collecting tomograms at four different sample-to-focus distances, which were then used for phase retrieval to generate 2D phase maps^[4]^. A total of 1500 projections were acquired for each scan.

**Figures**

**
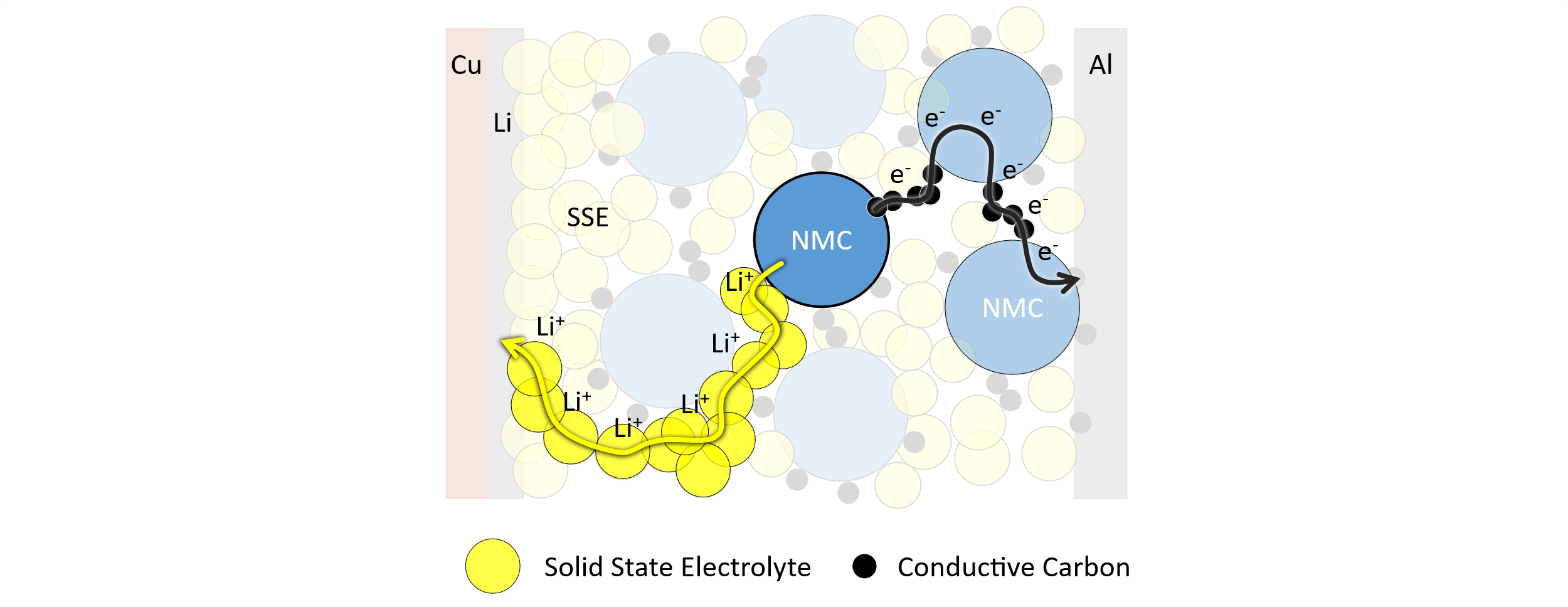
Figure S1.** Schematic illustration of a typical solid-state battery configuration.


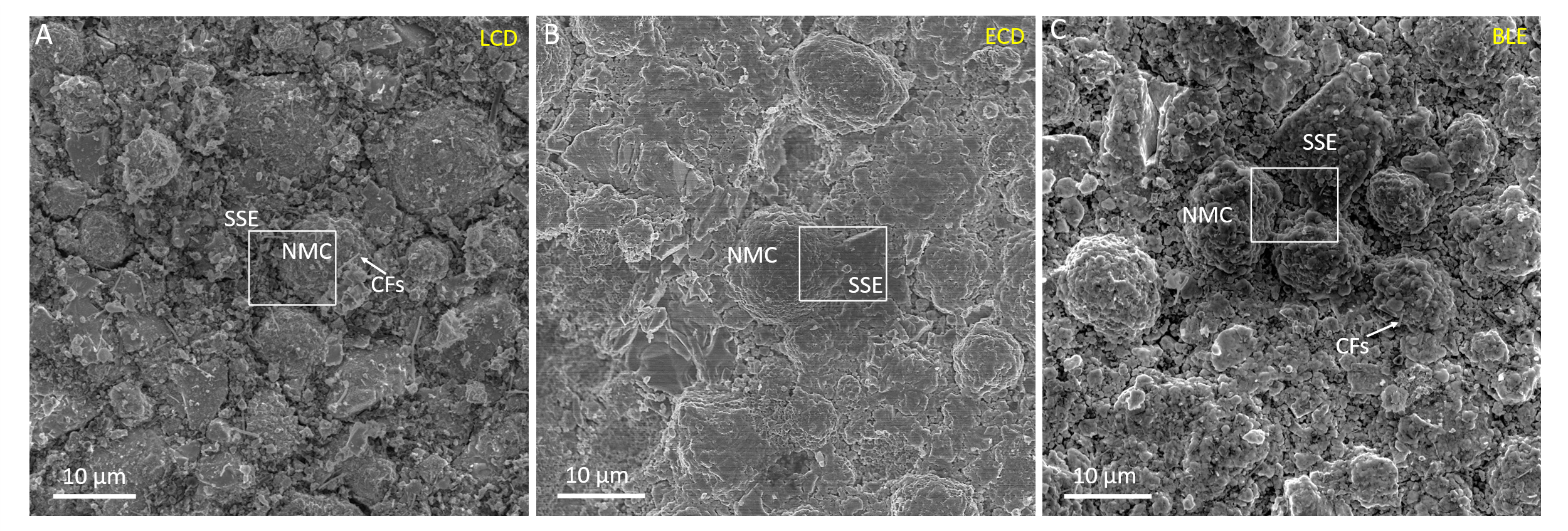


Figure S2. The top-down SEM images of surface morphologies of fabricated LCD (A), ECD (B), and BLE (C) electrodes.

**
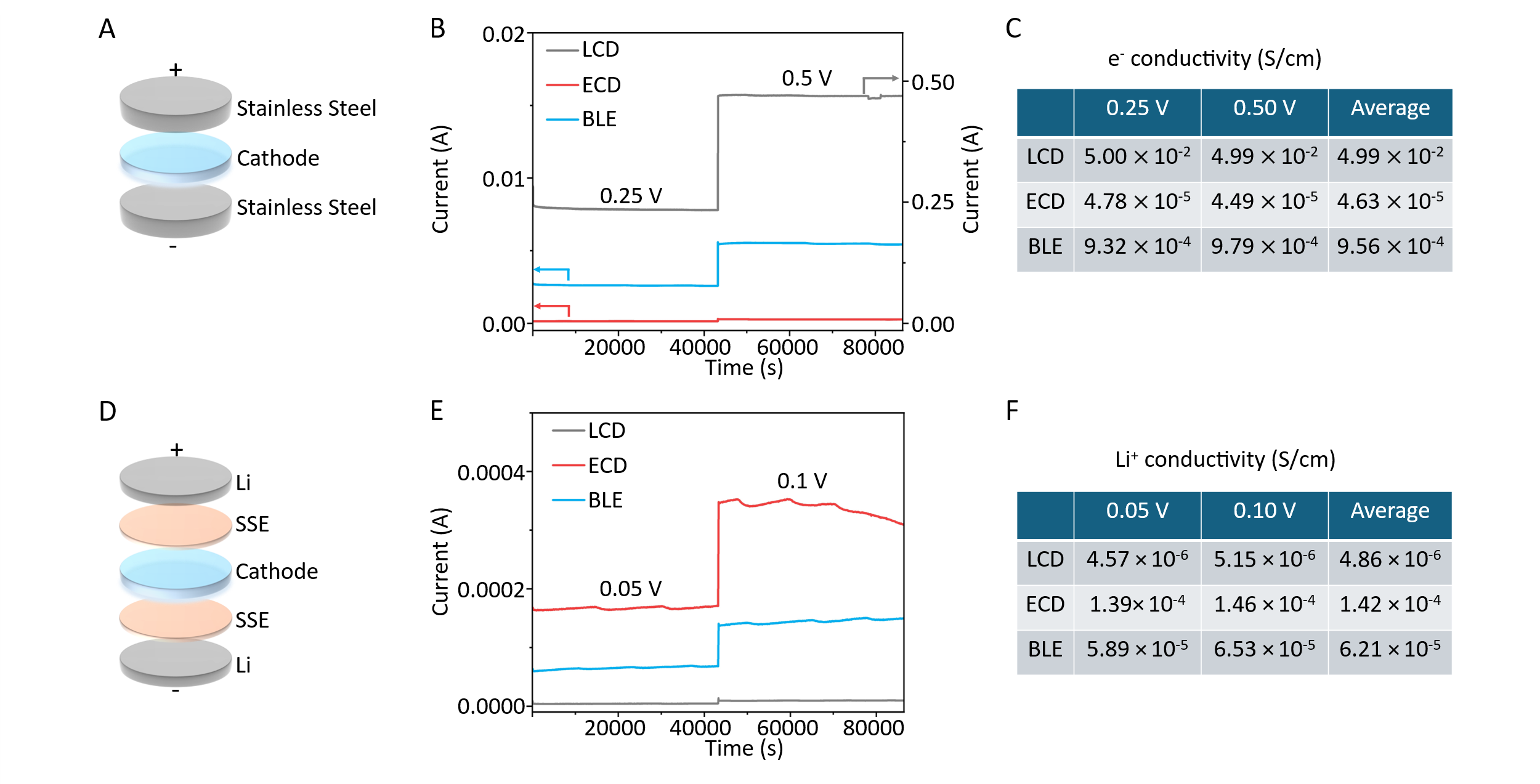
**

Figure S3. Electronic and ionic conductivities. (A) The schematic of ion-blocking cell; (B) The time-current response curve using ion-blocking cell; (C) e^-^ conductivity of LCD, ECD, and BLE; (D) The schematic of electron-blocking cell; (E) The time-current response curve using electron-blocking cell; (F) Li^+^ conductivity of LCD, ECD, and BLE.


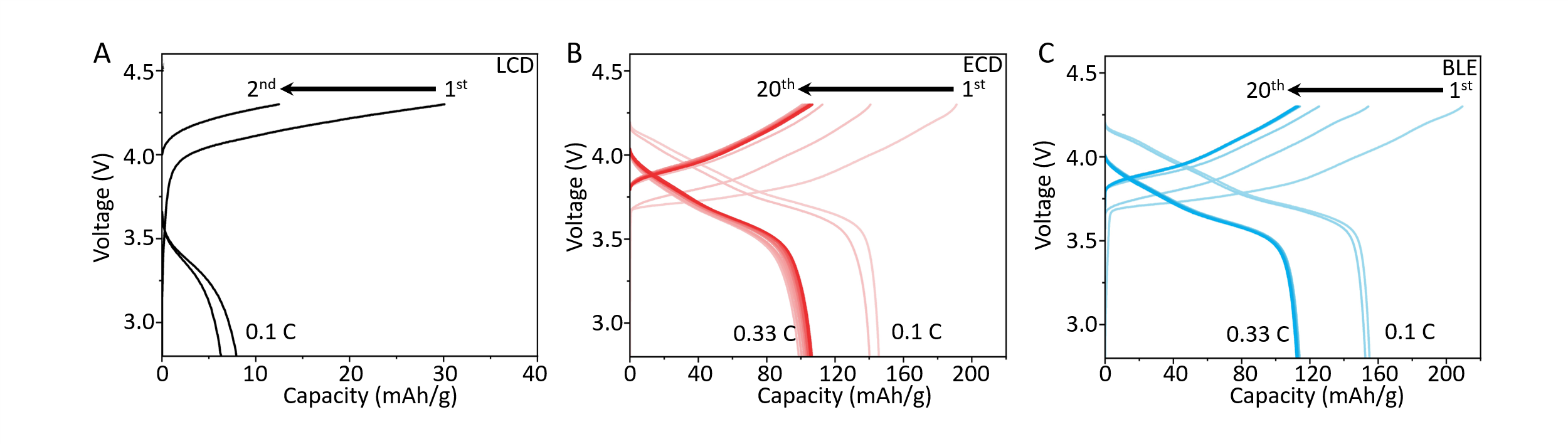
**Figure S4.** Charge-discharge profiles of LCD (A), ECD (B), and BLE (C). LCD shows almost no capacity at 0.33 C.


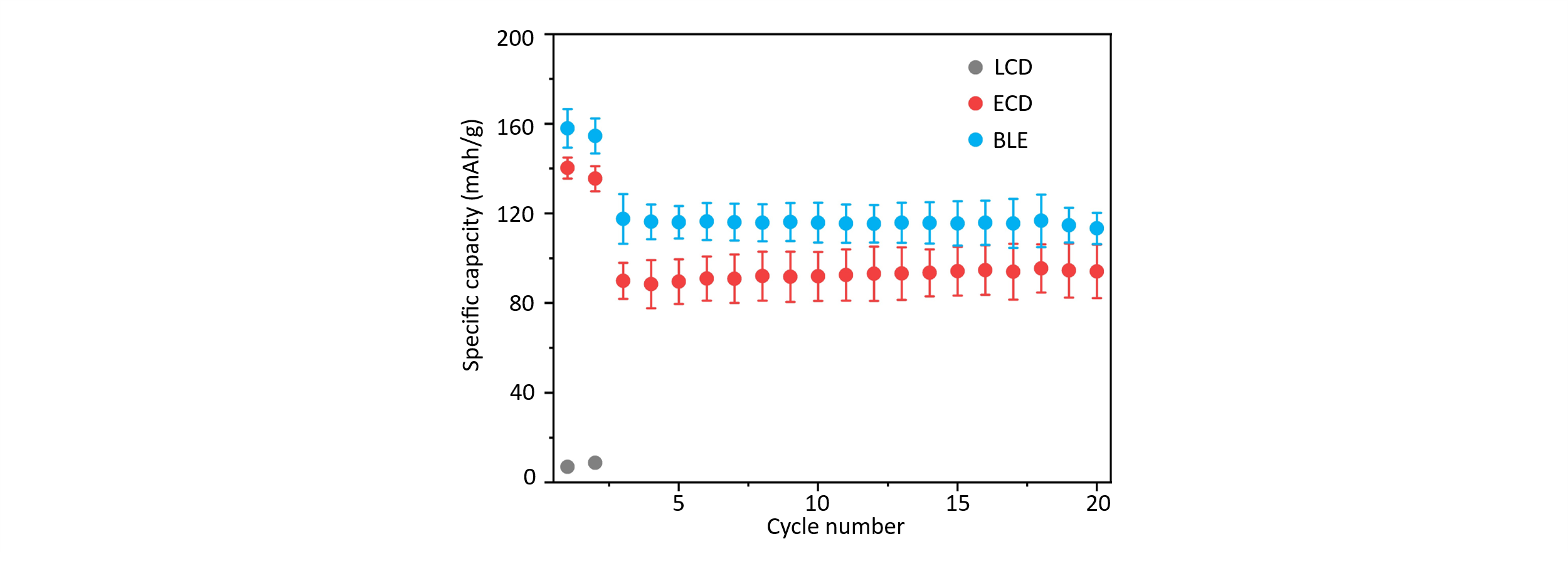


**Figure S5.** Cycling performance. LCD shows almost no capacity at 0.33 C. Error bars represent the mean ± standard deviation (n=3).

**
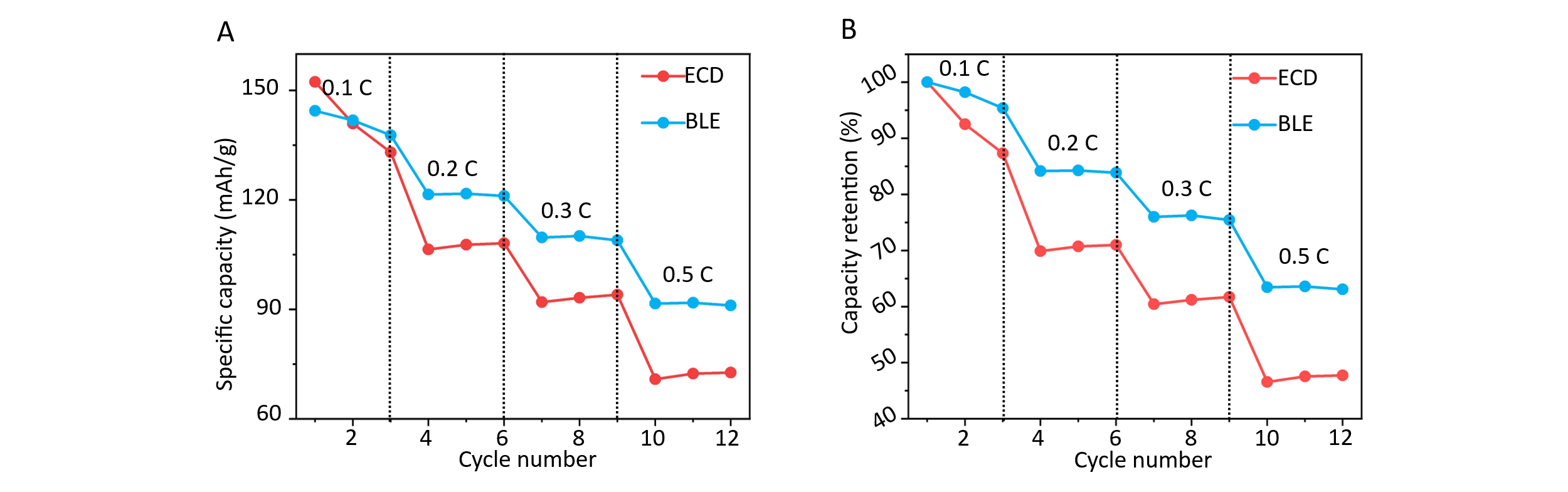
Figure S6.** (A) Rate performance of ECD and BLE; (B) Capacity retention.

**
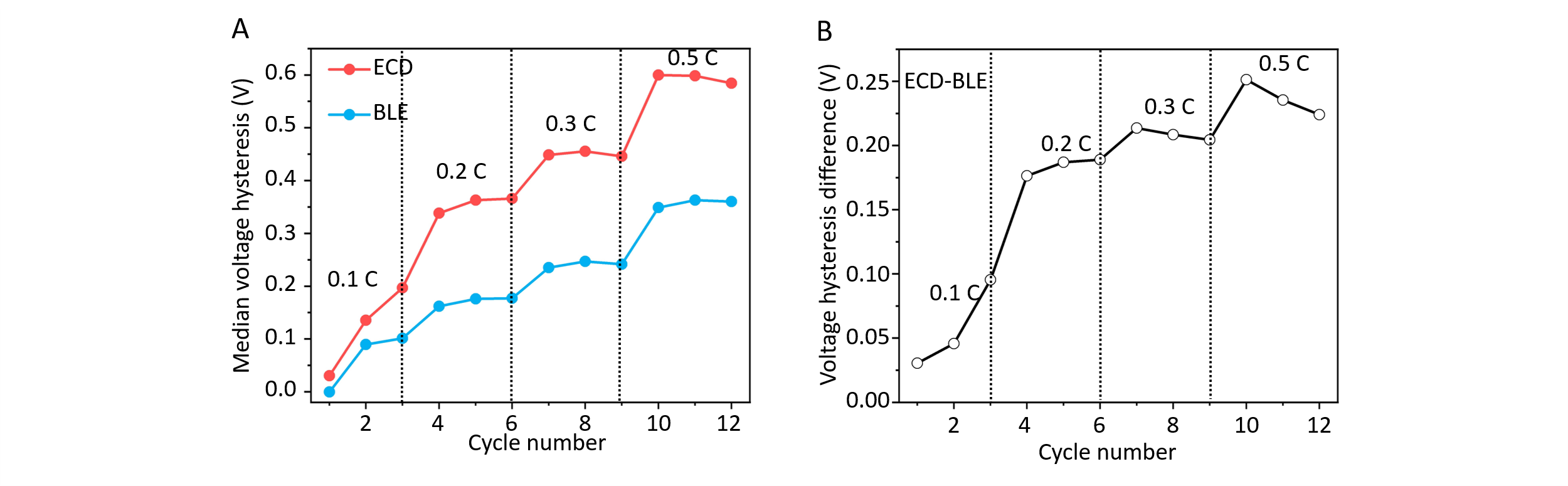
**

Figure S7. (A) Median voltage hysteresis in ECD and BLE; (B) The difference in median voltage hysteresis between ECD and BLE across different rates.


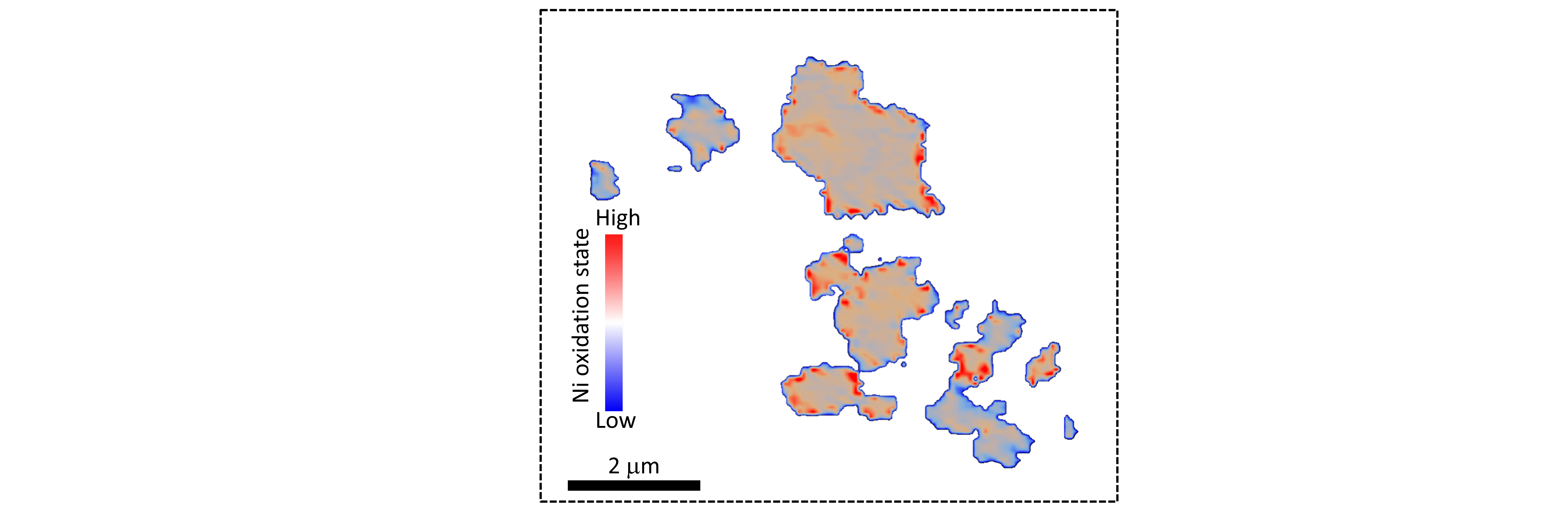


**Figure S8.** Ni oxidation state mapping within discharged primary particles of the cycled BLE cathode.


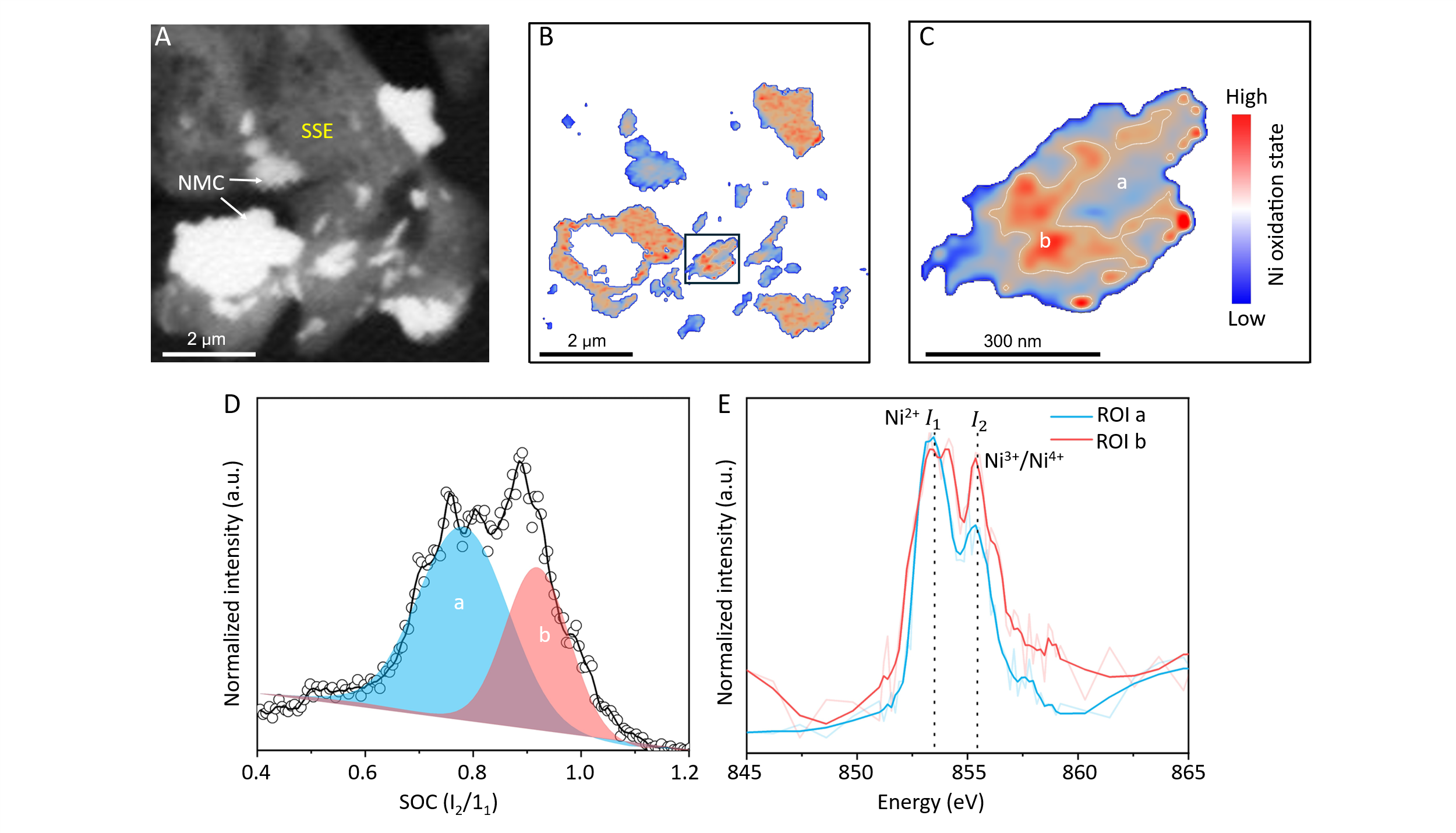


Figure S9. Charge distribution within primary particles of cycled ECD cathode. (A) STXM image of cycled ECD primary particles; (B) Ni oxidation state mapping within discharged primary particles of ECD; (C) Ni oxidation state mapping within a primary particle randomly selected from (B); (D) Probability distribution of Ni oxidation state within the selected primary particle; (E) Ni *L*_3_-edge XAS spectrum extracted from the regions of interest: a and b in (C).


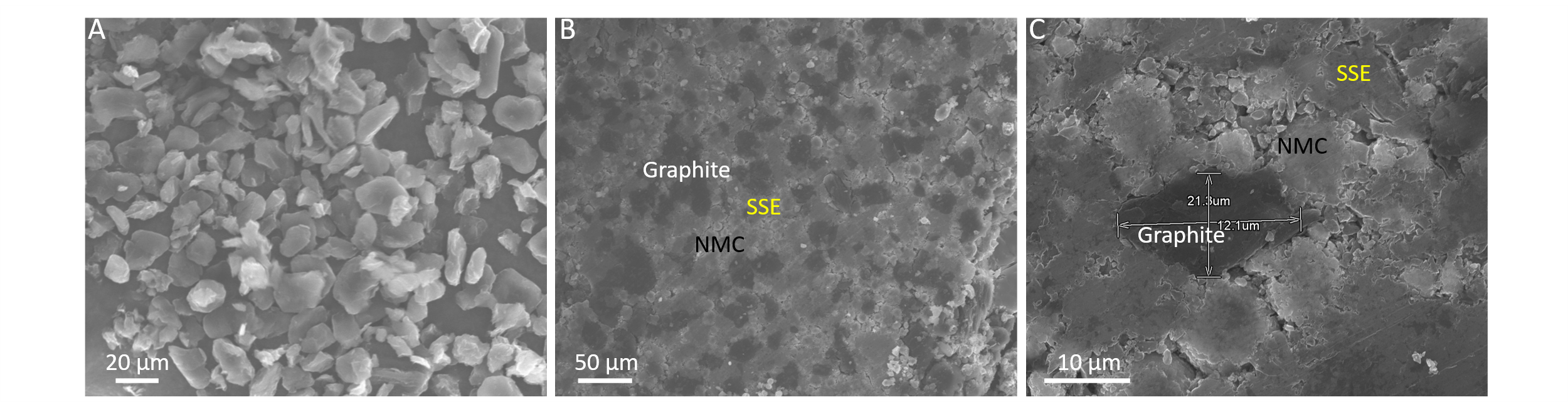


Figure S10. Micromorphology of cathode used in model system. (A) SEM image of graphite; (B and C) SEM images of top morphology of fabricated composite cathode.


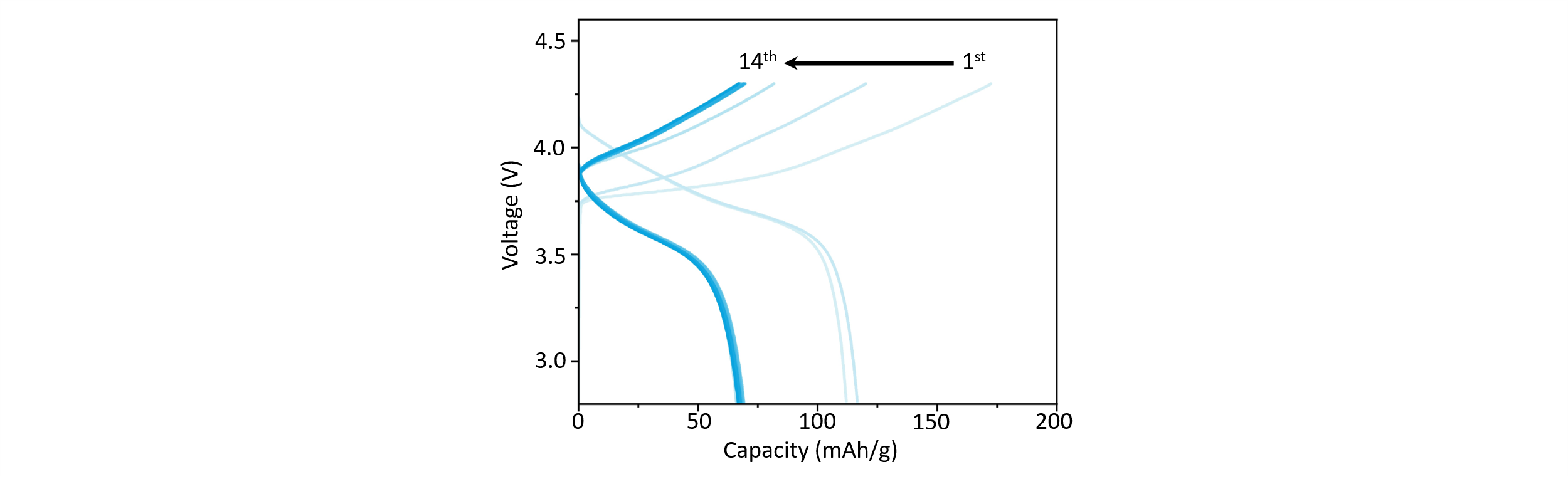


**Figure S11.** Charge-discharge profiles of model system.


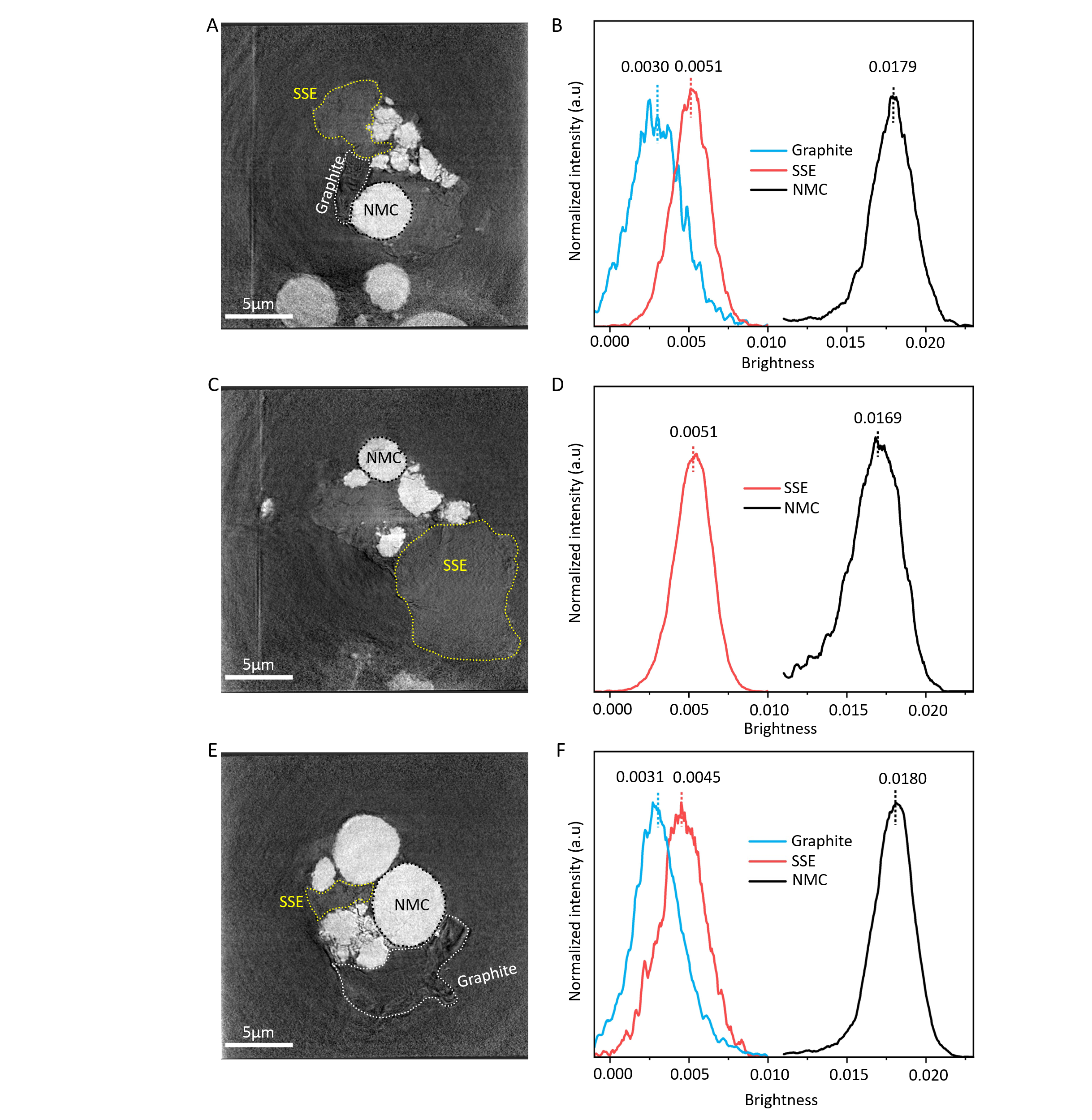


Figure S12. Selected cathode slices of model system with different micromorphology (A, C, and E) and the corresponding brightness profiles (B, D, and F) within selected regions of SSE, graphite, and NMC, respectively. (B) is extracted from (A); (D) is extracted from (C); (F) is extracted from (E).


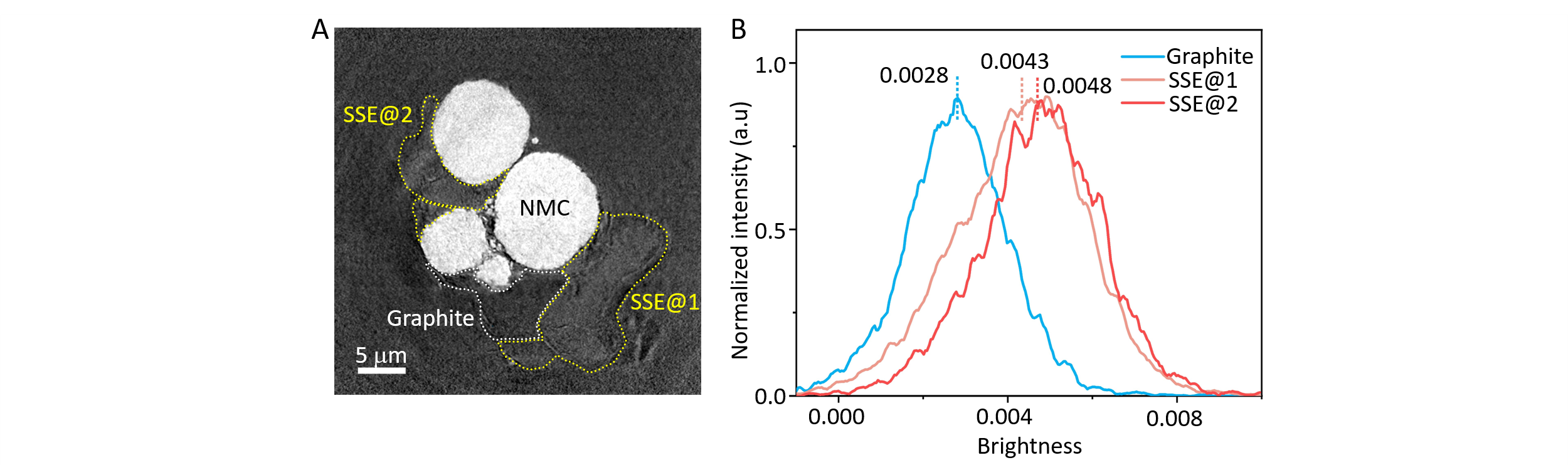


Figure S13. (A) Phase segmentation in a selected slice of model system used for further charge distribution analysis. NMC is easily identifiable, SSEs and graphite are distinguished by their brightness. (B) Brightness profiles of the three different regions.


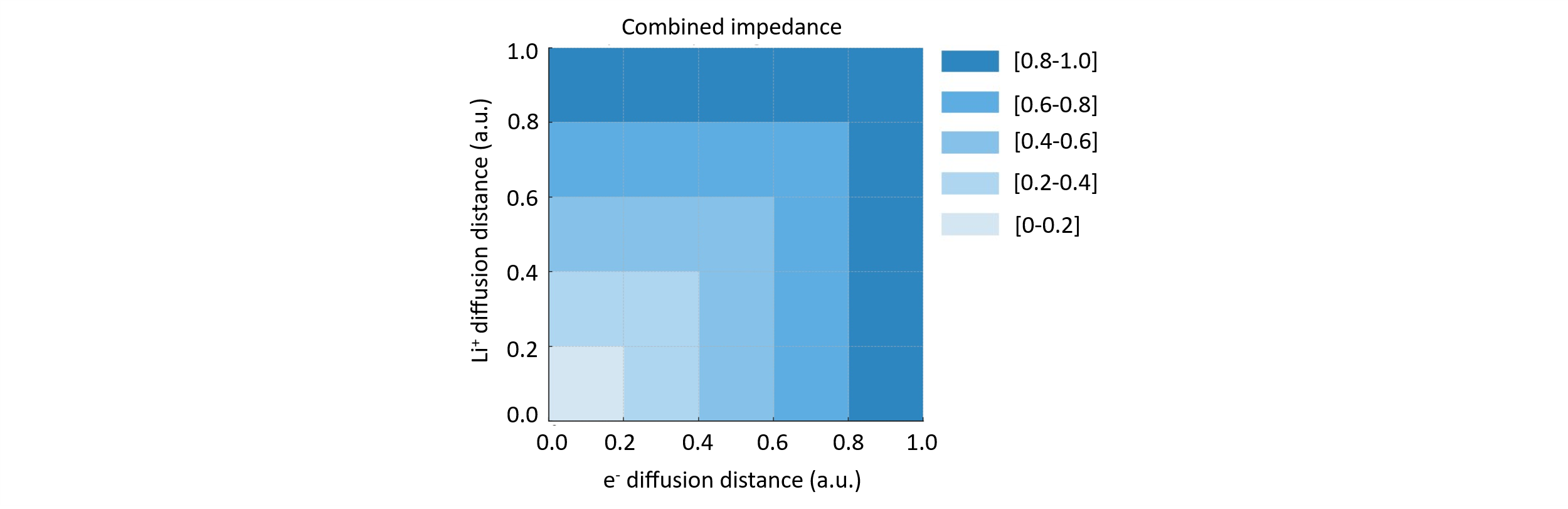


Figure S14. The combined impedance categorized into five regions: [0-0.2], [0.2-0.4], [0.4-0.6], [0.6-0.8], [0.8-1.0].


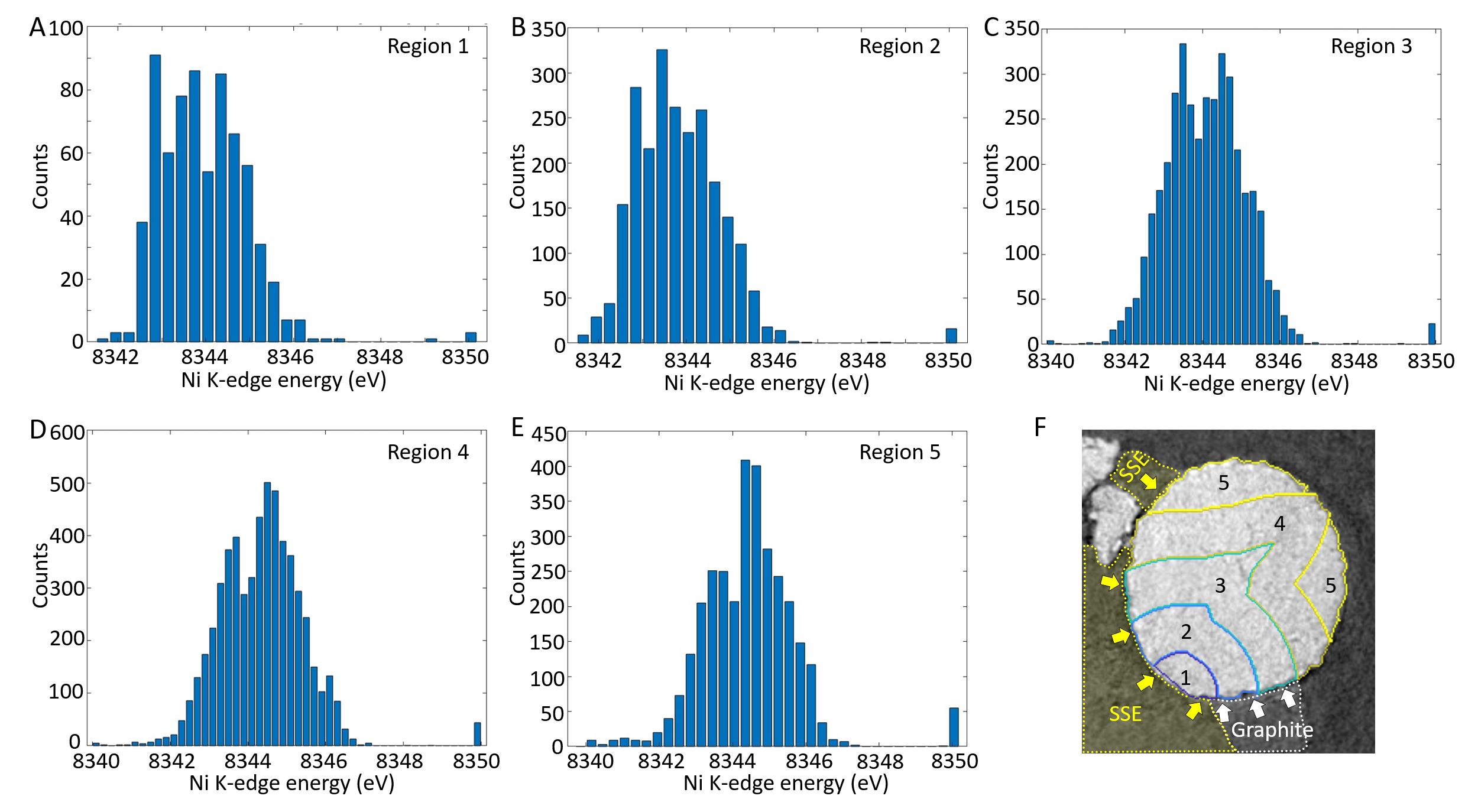


Figure S15. (A-E) Ni *K*-edge energy statistics for five regions with varying impedance in the diffusion distance model, considering both Li^+^ and e^-^ diffusion lengths from the SSE/graphite-to-NMC interface; (F) The five defined regions in the diffusion distance model, labeled as Regions 1 through 5, correspond to combined impedance ranges of [0-0.2], [0.2-0.4], [0.4-0.6], [0.6-0.8], and [0.8-1.0], respectively.


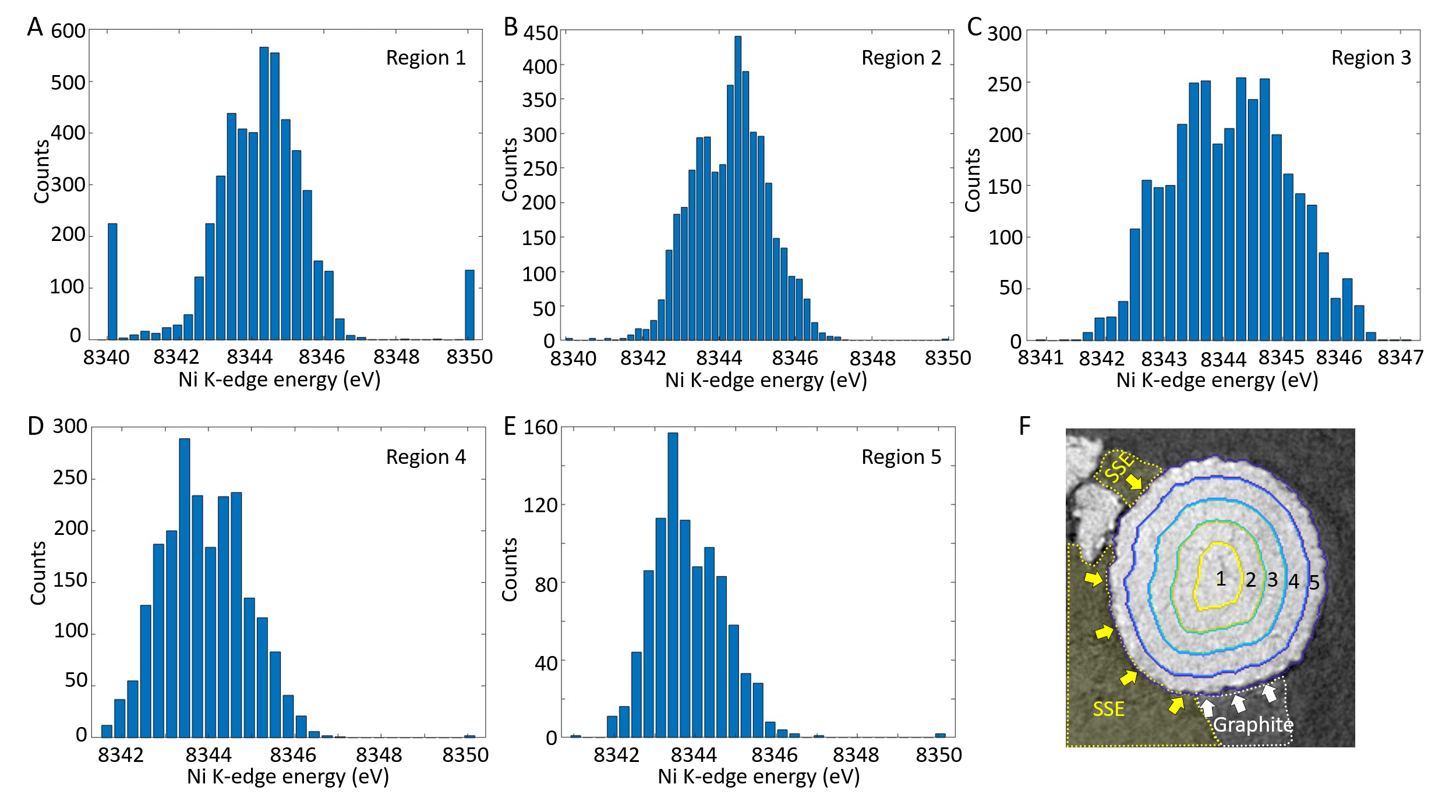


Figure S16. (A-E) Ni *K*-edge energy statistics for five regions with varying impedance in the core-shell model, based on both Li^+^ and e^-^ diffusion lengths starting from the NMC surface, without considering microenvironment differences; (F) The five defined regions in the core-shell model, labeled as Regions 1 through 5, correspond to combined impedance ranges of [0-0.2], [0.2-0.4], [0.4-0.6], [0.6-0.8], and [0.8-1.0], respectively.


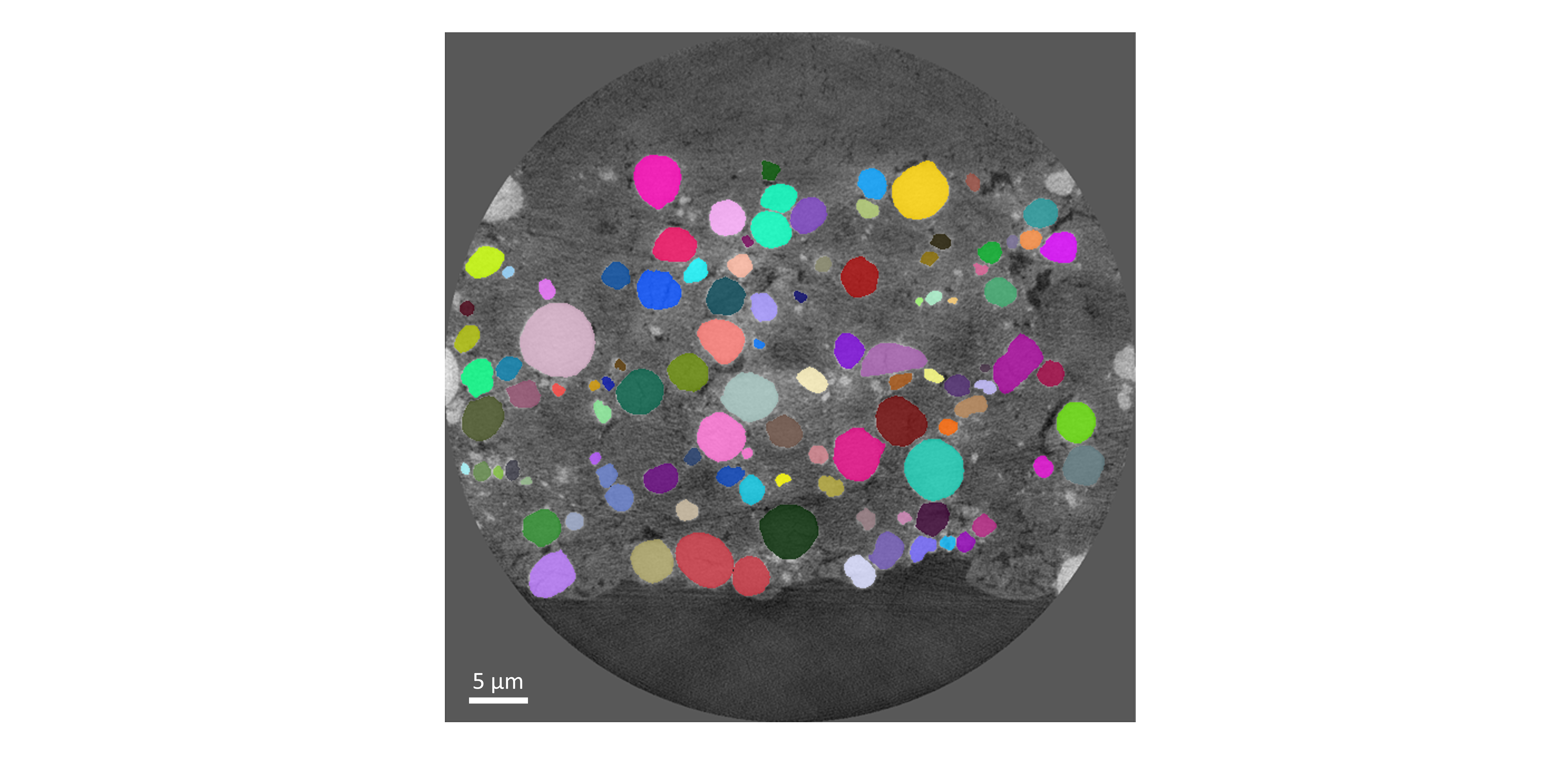


**Figure S17.** Labeled NMC particles from the nano-resolution hard X-ray phase-contrast holotomography data.

**References**

[1] Y. Wang, H. Hao, K. G. Naik, B. S. Vishnugopi, C. D. Fincher, Q. Yan, V. Raj, H. Celio, G. Yang, H. Fang, Y. M. Chiang, F. A. Perras, P. Jena, J. Watt, P. P. Mukherjee, D. Mitlin, *Adv. Energy Mater.* **2024**, *14*, 2304530.

[2] A. P. Hitchcock, *J. Electron Spectros. Relat. Phenomena* **2023**, *266*, 147360.

[3] Y. Liu, F. Meirer, P. A. Williams, J. Wang, J. C. Andrews, P. Pianetta, *J. Synchrotron Radiat.* **2012**, *19*, 281–287.

[4] P. Cloetens, W. Ludwig, J. Baruchel, D. Van Dyck, J. Van Landuyt, J. P. Guigay, M. Schlenker, *Appl. Phys. Lett.* **1999**, *75*, 2912–2914.
